# Elevational Adaptation in the Moth *Thitarodes xiaojinensis*: HSP60A‐Mediated Thermal Tolerance Evolution Under Gene Flow

**DOI:** 10.1002/ece3.74635

**Published:** 2026-11-09

**Authors:** Qiuyang Wei, Yuanchuan He, Shuai Pang, Shijiang Chen

**Affiliations:** ^1^ Chongqing University of Chinese Medicine Chongqing China; ^2^ Chongqing Academy of Chinese Materia Medica Chongqing China; ^3^ Glbizzia Biosciences Beijing China

**Keywords:** elevational adaptation, gene flow, *HSP60A*, population genomics, *Thitarodes xiaojinensis*

## Abstract

Climate warming intensifies thermal selection on high‐elevation species, yet the molecular mechanisms enabling rapid adaptation under ongoing gene flow remain poorly understood. We examined differentiation between High‐elevation (High) and Low‐elevation (Low) ecotype samples of *Thitarodes xiaojinensis*, an endemic ghost moth of the Qinghai‐Tibet Plateau, using SLAF‐seq, RNA‐seq profiling and survival experiments. Phylogenetic analyses confirm the two ecotype samples are conspecific, and they differ in forewing phenotype. Population structure at *K* = 2 uncovers elevation‐correlated clustering despite weak genome‐wide structure (optimal *K* = 1). Genome‐wide *F*_ST_ scans (Weir & Cockerham) identify HSP60A as a prominent outlier locus (*F*_ST_ = 0.391, top 1.4% of genome‐wide windows). Targeted PCR amplification and sequencing confirmed a nonsense mutation (Glu378*) in the *HSP60A* coding sequence. This derived allele was carried by 80% (4/5) of Low individuals and by none of 5 High individuals (0 of 10 chromosome copies, one‐sided 95% upper bound on allele frequency ≈ 26%), suggesting that it is associated with incipient ecological divergence between the two ecotypes. Co‐expression network analysis grouped the chaperone genes into two systems with divergent thermal responses. The small *HSPs* were induced 2.3‐fold from 8°C to 16°C, whereas the classical chaperones were not induced, and *HSP60A* was consistently placed within the latter (median biweight midcorrelation +0.56 with classical chaperones versus −0.27 with small *HSPs*, *p* = 3.5 × 10^−9^). Across the three temperatures the Low ecotype expressed *HSP60A* at lower levels than the High ecotype, significant under log_2_(TPM + 1) (*p* = 0.020) but not under the other two scales, and survived better at 16°C (*p* = 0.0342). These results link a coding variant at *HSP60A*, its lower transcript level in the Low ecotype and the better survival of that ecotype at 16°C, and identify *HSP60A* as a candidate locus for ecotype‐informed conservation of the *Ophiocordyceps sinensis* host insect.

## Graphical abstract

Using an integrative approach combining population genomics, transcriptomics, and thermal survival experiments, we identify HSP60A as a key adaptive locus in *Thitarodes xiaojinensis*—the obligate host of Ophiocordyceps sinensis—distributed across 3193–4285 m on the Qinghai‐Tibet Plateau. Despite ongoing gene flow, High‐ and Low‐altitude ecotypes show distinct thermal tolerance profiles, with Low ecotype individuals exhibiting reduced HSP60A expression yet enhanced survival at 16°C. These findings reveal how molecular adaptation can be maintained under gene flow in high‐altitude endemic species facing climate warming.

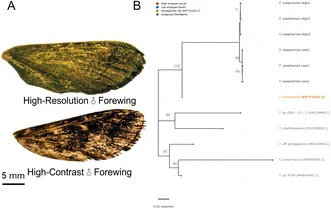

## 1 Introduction

Temperature shapes insect development, metabolism, and reproduction. Outside a species' viable thermal range, development slows, energy budgets contract, and survival declines (Sunday et al. 2011). Because every individual is subject to this constraint, temperature acts as a selective agent, and populations under different thermal regimes diverge in their thermal performance optima. Rising mean temperature therefore does more than shift a species' geographic range. By modifying the thermal environment experienced by local populations, it alters which individuals produce the greatest number of offspring and thereby reshapes local selection pressures (Hoffmann and Sgrò 2011). The Qinghai–Tibet Plateau (QTP) provides a natural system to investigate these evolutionary shifts in selection. The QTP is the highest and most extensive plateau on Earth, where steep elevational gradients compress broad climatic variation across short horizontal distances. In addition to this inherent elevational climatic heterogeneity, the QTP is warming at roughly 2.5 times the global average rate (Zhao et al. 2020), a trend that intensifies thermal selection on endemic organisms (Kattel 2022). As its endemic biota have diversified under prolonged isolation and ongoing warming reshapes plateau communities (Liao et al. 2023; Lang et al. 2025), understanding how these species adapt to temperature is therefore central to forecasting their persistence under continued climate warming.

The endemic moth *Thitarodes xiaojinensis* (Lepidoptera: Hepialidae), a principal host of the medicinal fungus *Ophiocordyceps sinensis*, provides a tractable system for exploring thermal adaptive evolution on the QTP. This species occupies alpine meadows from 3000 to 4800 m across the eastern plateau (Li et al. 2011). Because the fungus completes its life cycle only in the moth larva, the thermal limits of the moth also set the elevational range within which the *O. sinensis* harvest can be sustained. Exposed year‐round to alpine cold, this species may withstand low temperatures through trehalose metabolism, membrane lipid adjustment, and heat‐shock proteins that stabilize cell structures. Cold tolerance has been examined in the congeneric Thitarodes pui, where cold exposure shifts the expression of desaturase and heat‐shock genes within days and seasonal sampling shows the same genes tracking field temperature (Min et al. 2016, 2017; Zou et al. 2011). However, as the plateau has warmed, the two ecotype samples differ in forewing phenotype. High‐elevation individuals exhibit tawny forewings with diffuse dark markings, whereas low‐elevation conspecifics show a darker ground color with contrasting pale markings. The lower boundary of *O. sinensis* distribution has shifted upward by approximately 400 m since the late 20th century, whereas *T. xiaojinensis* has maintained its elevational range, indicating thermal niche stability (Chen et al. 2025). Genome‐wide analyses resolve the samples into elevational clusters despite weak genome‐wide structure and ongoing gene flow. Phenotypic and genetic differentiation associated with thermal adaptation has persisted, as it has in other alpine taxa (Danzey et al. 2024). How such differentiation translates into thermal performance can be assessed against the heat‐shock responses documented in other moths and insects.

In other moths, heat stress up‐regulates *Hsp70* in proportion to its intensity (Bahar et al. 2013; Zhao et al. 2025). This induction, however, carries fitness costs, and populations that are more heat‐tolerant tend to express less *Hsp70* at a given temperature, consistent with an elevated threshold for mounting the heat‐shock response (Sørensen et al. 2001). Heat‐shock responses have since been characterized in other Lepidoptera (Shen et al. 2014) and in other insect orders (Ware‐Gilmore et al. 2023). Across these groups, whole‐organism heat adaptation is attributed mainly to how the heat‐shock response is regulated, not to its absolute magnitude (Moseley 1997; Dündar et al. 2025). In most of these systems, however, thermal tolerance is inferred primarily from gene expression data, and the sequence variants that determine a population's response threshold are seldom identified. Whether high‐ and low‐elevation *T. xiaojinensis* ecotypes differ in *HSP* response magnitude or threshold, and what trade‐offs each strategy entails, remain open. This system, with phenotypic and thermal‐performance divergence maintained between the two *T. xiaojinensis* ecotypes, provides an opportunity to link sequence variation at specific loci to the regulatory networks in which they function.

Genome‐wide scans and expression profiling together can address these gaps. Correlating the two layers places a differentiated variant within a regulatory network, something neither approach achieves alone. In this study we combine population genomics, transcriptomics, and thermal‐tolerance phenotyping in the two *T. xiaojinensis* ecotypes. We aim to (1) characterize population structure and gene flow, (2) identify genome‐wide outlier loci, (3) relate differentiated loci to thermal‐stress transcriptional responses and co‐expression networks, and (4) link elevational gradients to ecotypic differences in thermal tolerance. This work addresses how divergence persists despite ongoing gene flow and provides a framework for assessing how alpine endemics may respond as the climate warms.

## 2 Materials and Methods

### 2.1 Sample Collection and Processing

Two ecotype samples were collected from Xiaojin County, Sichuan Province, China, the Low ecotype from Chongde Township (102°19′9″ E, 31°12′55″ N, 3193 m a.s.l.) and the High ecotype from Meiwo Township (102°21′54″ E, 30°47′29″ N, 4285 m a.s.l.). The larvae were transferred to the laboratory, where each ecotype was maintained as a laboratory population under identical controlled conditions, constant 8°C, continuous darkness (0 L:24D) and 75% relative humidity, for at least two generations. The two ecotype samples were reared in separate containers and were never crossed, so that the contrast between them reflects field origin rather than laboratory mixing. To minimize maternal and environmental effects, newly hatched second‐generation larvae were then selected at the start of the experiment and reared individually for 4 months as a pre‐treatment acclimation, one per cylindrical glass jar (8 cm in diameter and 7 cm in height) and each given 10 g of *Polygonum viviparum* rhizome together with 10 g of *Potentilla anserina* root at the start of each rearing period. This ration was sufficient for the entire period and was not replenished. The diet and the rearing vessels were identical before and after the temperature treatment, and at the start of each temperature treatment every larva was transferred to a fresh jar with fresh diet. The temperature treatments were carried out under the same environmental conditions as the pre‐treatment rearing, with continuous darkness and 75% relative humidity maintained throughout, so that temperature was the only variable that differed among treatments. Fourth‐instar larvae at the stage of vigorous growth were used in all experiments.

### 2.2 COI Sequencing and Phylogenetic Analysis

Specimens were identified by COI before any between‐sample comparison, since all downstream comparisons depend on correct species assignment. A fragment of the mitochondrial cytochrome c oxidase subunit I (COI) gene was PCR‐amplified from three individuals per ecotype using universal primers, yielding amplicons of 326–335 bp. After trimming 27–34 bp of 5′ primer and adapter residues, a 298‐bp conserved coding region was retained. Sequences were aligned with the conspecific GenBank reference KP772242.1, and pairwise divergences were estimated both as *p*‐distances and under the Kimura two‐parameter (K2P) model. A neighbor‐joining tree was constructed with 1000 bootstrap replicates and rooted using five congeneric outgroup taxa (Chen et al. 2017).

### 2.3 SLAF‐Seq Library Construction and SNP Discovery

SLAF‐seq was chosen because it enables reference‐free genome‐wide SNP genotyping in non‐model species, so that outlier loci resistant to gene‐flow‐driven homogenization can be screened without prior genomic resources (Li et al. 2023; Mamat et al. 2023). It shares the two‐enzyme, size‐selection design of double‐digest RADseq (Peterson et al. 2012) but derives the enzyme pair and fragment size range from in silico genome digestion, yielding more scorable loci with a more uniform genomic distribution. Genome‐wide molecular markers were developed using SLAF‐seq technology in 35 *T. xiaojinensis* samples (19 individuals from the Low‐elevation ecotype and 16 individuals from the High‐elevation ecotype). Dual‐enzyme digestion (HaeIII + HinCII) was selected based on in silico genome digestion predictions, with SLAF tags defined as fragments of 314–364 bp to avoid repetitive regions and ensure uniform distribution. Genomic DNA was digested, followed by 3′‐adenylation, adapter ligation, PCR amplification, purification, and gel electrophoresis for fragment size selection. Libraries were sequenced on an Illumina platform with paired‐end 126 bp reads (Kozich et al. 2013). Clean reads (adapter‐trimmed, Q20 ≥ 85%, minimum length 50 bp) were aligned to the *T. xiaojinensis* reference genome using BWA‐MEM (v0.7.17; Li and Durbin 2009). SNPs were identified via a combined pipeline of GATK HaplotypeCaller (v4.1) in gVCF mode (McKenna et al. 2010) and SAMtools mpileup (v1.15) (Li et al. 2009), retaining sites with minimum depth ≥ 3× and maximum depth ≤ 200×.

### 2.4 Population Genetic Structure and Genome‐Wide Differentiation Analysis

Structure and differentiation were each assessed by more than one method, so that the inference does not rest on a single statistic. High‐confidence SNPs were defined by minor allele frequency (MAF ≥ 0.05) and genotyping completeness (call rate ≥ 70% per individual, missing rate ≤ 30% per site). Population structure was assessed with ADMIXTURE (v1.22) for *K* = 1–10 using five‐fold cross‐validation repeated 10 times, the optimal *K* being the value that minimized the CV error. Phylogenetic relationships were inferred by neighbor joining in MEGA X with 1000 bootstrap replicates, and pairwise kinship matrices were calculated in GCTA (v1.92.1) and displayed as correlation heatmaps (Yang et al. 2011). *F*_ST_ and nucleotide diversity (π) were computed in VCFtools (v0.1.15) across 100‐kb sliding windows with a 10‐kb step (Danecek et al. 2011). Window *F*_ST_ was obtained as the Weir and Cockerham (1984) ratio of averages over all segregating sites in a window, *Σa*/(*Σa* + *Σb* + *Σc*), rather than as the mean of per‐site estimates, so that windows of differing marker density are placed on a common scale. An empirical null was generated by permuting the ecotype labels among the 35 individuals 999 times and recomputing window FST on each permuted genotype matrix. Window precision was quantified by a moving‐block bootstrap, in which the 10‐kb segments constituting a window were resampled with replacement 2000 times and the ratio‐of‐averages *F*_ST_ recomputed for each replicate. All window and resampling statistics were based on the filtered SLAF SNP set (Evo.recode.vcf; 1,637,482 sites at MAF ≥ 0.05; Low *n* = 19, High *n* = 16). Outlier thresholds were the 95th and 99th percentiles of the genome‐wide window *F*_ST_ distribution; windows carrying too few segregating sites for a stable FST estimate (π < 0.5 × 10^−4^ in either ecotype) were excluded when defining the 99th‐percentile threshold. Because the windows overlap, between‐ecotype π was additionally compared on a non‐overlapping subset (one window per 100 kb) using the Wilcoxon signed‐rank test.

### 2.5 *HSP60A* Candidate Variant Identification and Validation

Because the candidate site falls at the boundary of a SLAF tag and was absent from the filtered SNP set, we called its genotypes directly using aligned reads from the 35 individuals. All reads spanning this site were tallied. A single spanning read allowed genotype scoring for an individual, while samples with no recovered spanning reads were noted to have missing coverage at this locus. The region encoding position 378 of *T. xiaojinensis HSP60A* (Chr04.g05884.m1) was compared with ten additional lepidopteran *HSP60* orthologues spanning seven families, with the vertebrate orthologue *Homo sapiens* *HSPD1* (P10809.2), and with three more distantly related chaperonin‐60 family members, two of which are annotated as 63 kDa mitochondrial chaperonin‐like (Table S1). Positions were numbered according to T. xiaojinensis *HSP60A*, the equivalent residue in each sequence being assigned from the alignment with the conserved YEKEK motif (positions 383–387) as the anchor; a 31‐residue window (positions 363–393) was used to assess whether position 378 tolerates substitution among orthologues, and the ancestral state at this position was inferred parsimoniously from the outgroup sequence.

Five larvae were drawn at random from each of the two ecotype samples, independently of the individuals used for SLAF‐seq (five per ecotype; total *n* = 10). Genomic DNA was extracted by homogenizing samples in 1.5 mL microcentrifuge tubes with NP40 lysis buffer (Proteinase K to NP40 ratio 1:19, v/v), followed by incubation at 55°C for 24 h. The supernatant was collected after centrifugation (12,000g, 5 min) and heat‐inactivated at 98°C for 10 min. PCR amplification was performed in a 50 μL reaction containing 25 μL Taq mix buffer, 2.5 μL each of primers JD‐F (5′‐CATTGATGTTGAAGGGTGGGTGTTA‐3′) and JD‐R (5′‐CAGTACTAGGACTACTCTCCATCCA‐3′), 2 μL template DNA, and 18 μL ddH_2_O. The PCR program was: initial denaturation at 94°C for 3 min; 35 cycles of 94°C for 30 s, 58°C for 30 s, 72°C for 30 s; final extension at 72°C for 5 min; and indefinite hold at 12°C. Amplicons were purified and Sanger‐sequenced (ABI 3730xl) in both directions. Chromatograms were manually inspected and aligned using MUSCLE to identify the target SNP. Because no High‐elevation amplicon carried the derived allele, the one‐sided 95% upper bound on its frequency in this ecotype was obtained from the binomial distribution as 1–0.05^(1/*n*), where n is the number of sequenced chromosome copies (*n* = 10; five individuals).

### 2.6 Transcriptomic Sequencing and Analysis

RNA‐seq was used to capture genome‐wide expression dynamics under thermal stress and thereby link genomic variation to phenotypic change (Li et al. 2025). Three temperatures (8°C, 12°C and 16°C) spanned the natural range encountered by the two ecotypes. Larvae were reared individually in glass jars, with 30 larvae forming one replicate group and three biological replicates per ecotype per temperature (18 experimental units in total, 2 ecotypes × 3 temperatures × 3 replicates). Mortality was quantified at the end of the one‐month treatment period. Three surviving larvae were then taken at random from each replicate and pooled for total RNA extraction. Total RNA was extracted from whole larvae and checked by agarose gel electrophoresis, Nanophotometer spectrophotometry (OD260/280 and OD260/230 ≥ 1.8) and an Agilent 2100 Bioanalyzer (RNA Integrity Number ≥ 7.0). PolyA‐enriched mRNA was fragmented in NEB Fragmentation Buffer, reverse‐transcribed with random hexamers and ligated to dual‐index adapters. Libraries were quantified by Qubit fluorometry and the Agilent 2100 Bioanalyzer, normalized to 2 nM and sequenced on an Illumina platform with paired‐end 150 bp reads. Raw reads were quality‐filtered with FastQC, aligned to the reference genome with STAR (v2.7.10a) and quantified with featureCounts.

### 2.7 RNA‐Seq‐Based Co‐Expression Analysis

Co‐expression analysis used all 18 samples and the 11,046 genes with log_2_(TPM + 1) ≥ 1 in at least 6 samples. A signed hybrid network was built with WGCNA (Langfelder and Horvath 2008; Karimi‐Fard 2026; Ma et al. 2023; Zhao et al. 2024) on the topological overlap matrix, with the soft‐thresholding power chosen by the scale‐free topology criterion. Dynamic tree cutting (deepSplit = 2, minimum module size 30) defined the modules, merging those with an eigengene dissimilarity below 0.25. Module eigengenes were correlated with temperature and the binary ecotype label (High = 0, Low = 1). Module membership (kME) denotes the correlation between a gene and its module eigengene, whereas gene significance (GS) represents the correlation between a gene and the tested trait. Both metrics were calculated in this study, and gene significance was reported for assay temperature.

Chaperone sets were defined from functional annotation (Table S2), the classical set as canonical *HSP70*, *HSP90*, *DnaJ*/*HSP40* and chaperonin (TCP‐1/cpn60) members (*n* = 42, of which 30 were canonical) but excluding entries annotated only as chaperone‐binding, histone, tubulin‐specific or copper chaperones, and the sHSP set as Hsp20/α‐crystallin members (*n* = 21). Over‐representation in each module was tested by the hypergeometric distribution against permuted module labels. Temperature induction, the log_2_ fold change from 8°C to 16°C, was compared between sets by Mann–Whitney *U* tests on raw counts and after DESeq2 size‐factor correction (Love et al. 2014), and between‐ecotype differences at 16°C were expressed relative to the genome‐wide background and tested by two‐way ANOVA.

Pairwise biweight midcorrelation (bicor) was computed between *HSP60A* and every member of each set on log_2_(TPM + 1). Because module assignment and connectivity are scale‐dependent, all family‐level and target‐gene contrasts were repeated under log_2_(CPM + 1) and variance‐stabilized counts, with the scale stated for each contrast. The HSP60A‐centred network was drawn in Cytoscape v3.10, keeping edges with TOM ≥ 0.1 and |r| ≥ 0.85 (Ballouz et al. 2015).

## 3 Results

### 3.1 Ecotype Identification of *T. xiaojinensis*

Morphological comparison distinguished the two ecotypes (Figure 1A). In the neighbor‐joining tree of the 298‐bp COI region, each ecotype formed a monophyletic cluster (Figure 1B), and both clusters grouped with the conspecific reference KP772242.1, identifying all six sequences as *T. xiaojinensis*. Sequence divergence (Table 1) revealed 12 variable sites defining three ecotype‐specific haplotypes, with no haplotypes shared between ecotypes. Hap1 was recovered from all Low ecotype individuals (L1–L3), whereas Hap2 (H1) and Hap3 (H2, H3) represented the High ecotype. Mean p‐distance was 0.00% within the Low ecotype and 0.89% within the High ecotype, versus 3.58% between ecotypes (K2P = 3.67%). Divergence from the conspecific GenBank reference KP772242.1, obtained from a different collecting site in the same county, exceeded the divergence between the two ecotypes (*p*‐distance 5.29% and K2P 7.41% vs. 3.58% and 3.67%), yet remained below the congeneric interspecific distances (mean 9.98%), indicating deep intraspecific mitochondrial structure in this species.

**FIGURE 1:**
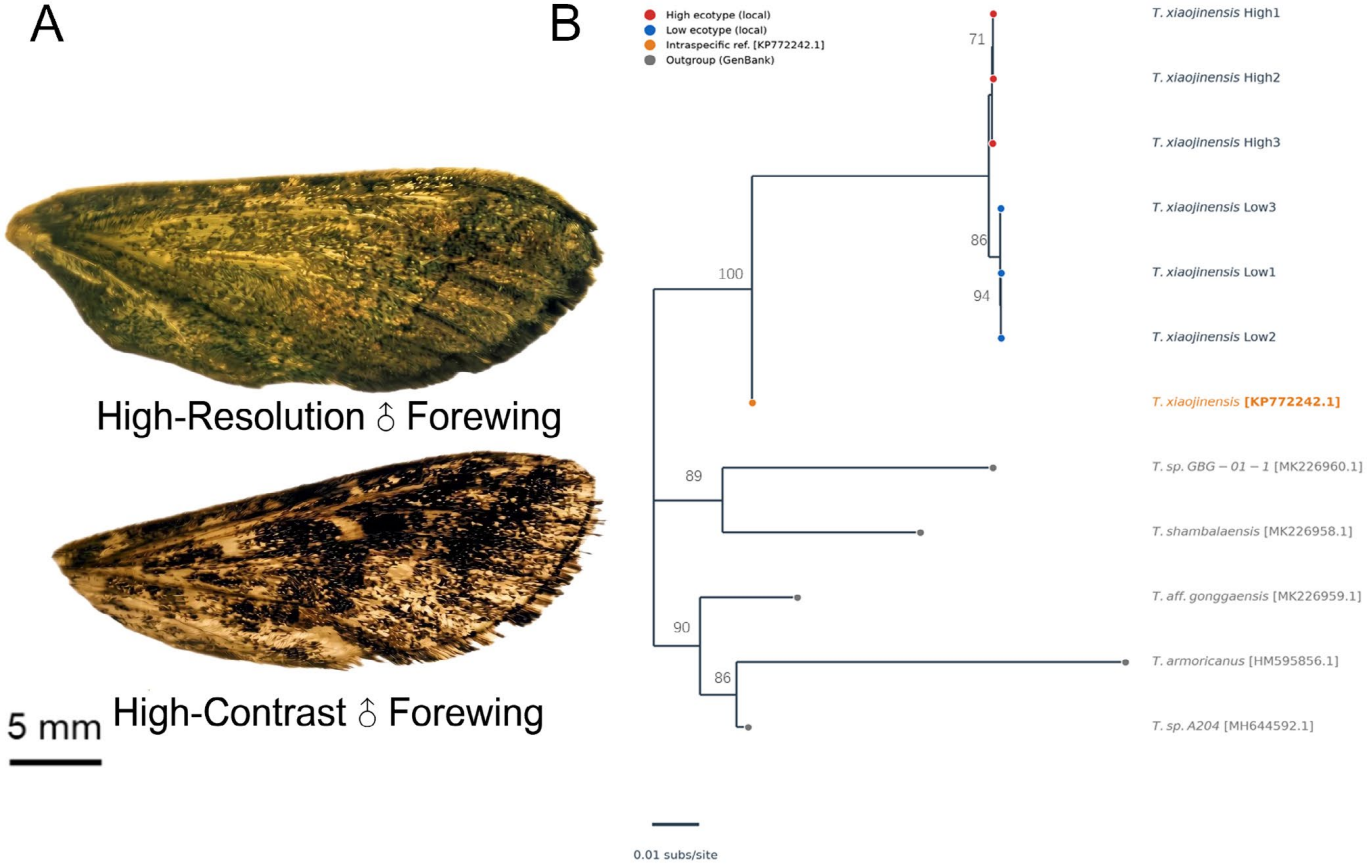
Forewing morphology and phylogenetic relationship of two male ecotypes of *T. xiaojinensis*. (A) Representative male forewing phenotypes. High ecotype (top) and Low ecotype (bottom). Scale bar = 5 mm. (B) Neighbor‐joining phylogenetic tree. The tree was inferred from the 298‐bp conserved COI region under the Kimura two‐parameter (K2P) distance model and rooted using five congeneric outgroup taxa (*T*. sp. GBG‐01‐1 [MK226960.1], *T. shambalaensis* [MK226958.1], *T. aff. gonggaensis* [MK226959.1], *T. armoricanus* [HM595856.1], *T*. sp. A204 [MH644592.1]). Bootstrap values (1000 replicates) are shown at internal nodes (values < 50 not displayed). Branch lengths are proportional to the number of substitutions per site (scale bar = 0.01 substitutions per site).

**TABLE 1:** COI sequence divergence among six local sequences of *T. xiaojinensis* and the GenBank reference KP772242.1 (298 bp, 12 variable sites).

Comparison	*n*	Hap.	*p*‐dist (%)	K2P (%)
Within Low (Hap1: L1/L2/L3)	3	1	0.00	0.00
Within High (Hap2: H1; Hap3: H2/H3)	3	2	0.89	0.90
Between ecotypes	6	3	3.58	3.67
Local vs. KP772242.1	6	3	5.29 (5.20–5.37)	7.41 (7.29–7.54)

### 3.2 Development of SLAF Tags and SNP Markers

Based on SLAF sequencing of the two ecotype samples (35 individuals), a total of 650,164 high‐quality SLAF tags were developed after quality control and filtering, with an average sequencing depth of 10.40× per individual. Among these, 438,617 tags were polymorphic, yielding 3,903,703 SNP markers distributed across all chromosomes for subsequent genetic diversity analysis (Figure 2).

**FIGURE 2:**
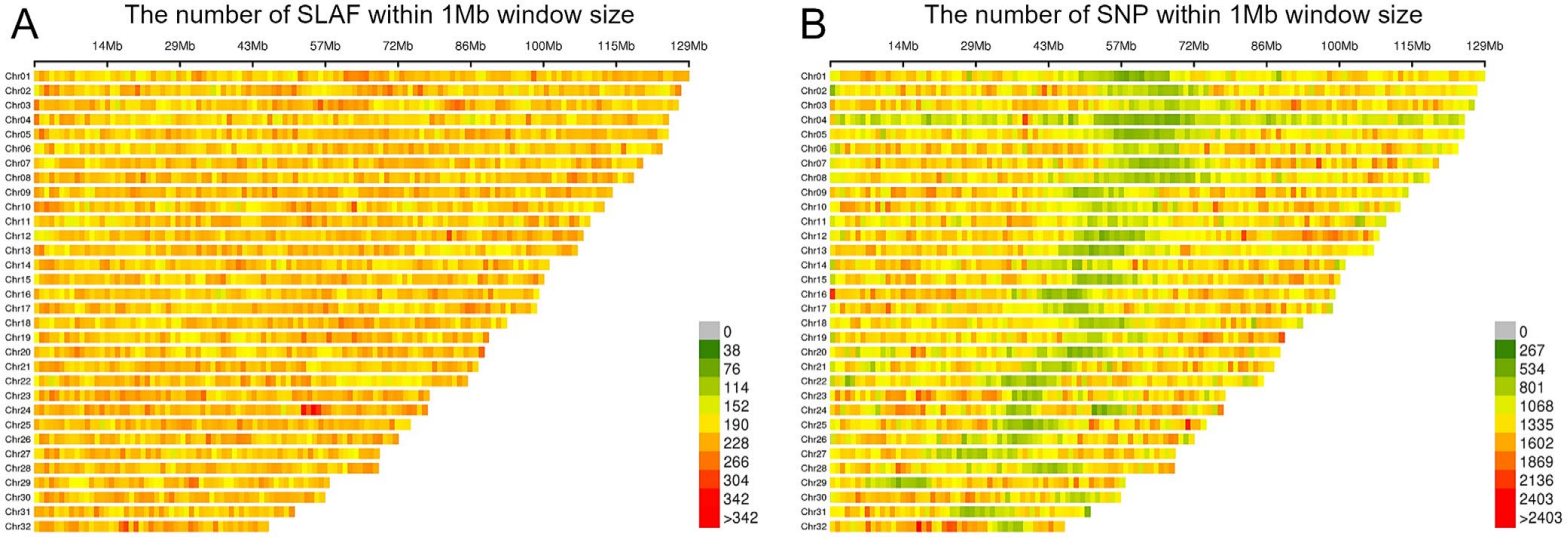
Chromosomal distribution of SLAF tags and SNPs in *T. xiaojinensis*. (A) Density of SLAF tags across chromosomes. Each horizontal bar represents one chromosome, divided into non‐overlapping 1‐Mb windows. The *x*‐axis indicates chromosome length (Mb); the color scale represents the number of SLAF tags per window (green, few; red, many). (B) Density of genome‐wide SNPs across chromosomes. Each horizontal bar represents one chromosome, divided into non‐overlapping 1‐Mb windows. The *x*‐axis indicates chromosome length (Mb); the color scale represents the number of SNPs per window (green, few; red, many).

### 3.3 Population Structure and Genome‐Wide Differentiation Analysis

Population genetic structure was assessed in the 35 individuals (19 Low, 16 High) by testing *K* = 1–10 in ADMIXTURE. The cross‐validation error was minimized at *K* = 1, indicating that a single‐population model best fits the genome‐wide data and hence only weak genome‐wide structure (Figure 3A). At *K* = 2, however, the individuals partitioned bimodally by elevational origin (Figure 3B). The genome‐wide neighbor‐joining tree and the pairwise kinship matrix recovered the same pattern, with within‐ecotype relatedness exceeding cross‐ecotype relatedness and the Low‐ecotype individuals forming a distinct cluster (Figure 3C,D). The *K* = 2 partition therefore captures an ecological signal superimposed on an otherwise weakly structured genome, a pattern characteristic of early‐stage ecotypic differentiation rather than speciation‐level divergence.

**FIGURE 3:**
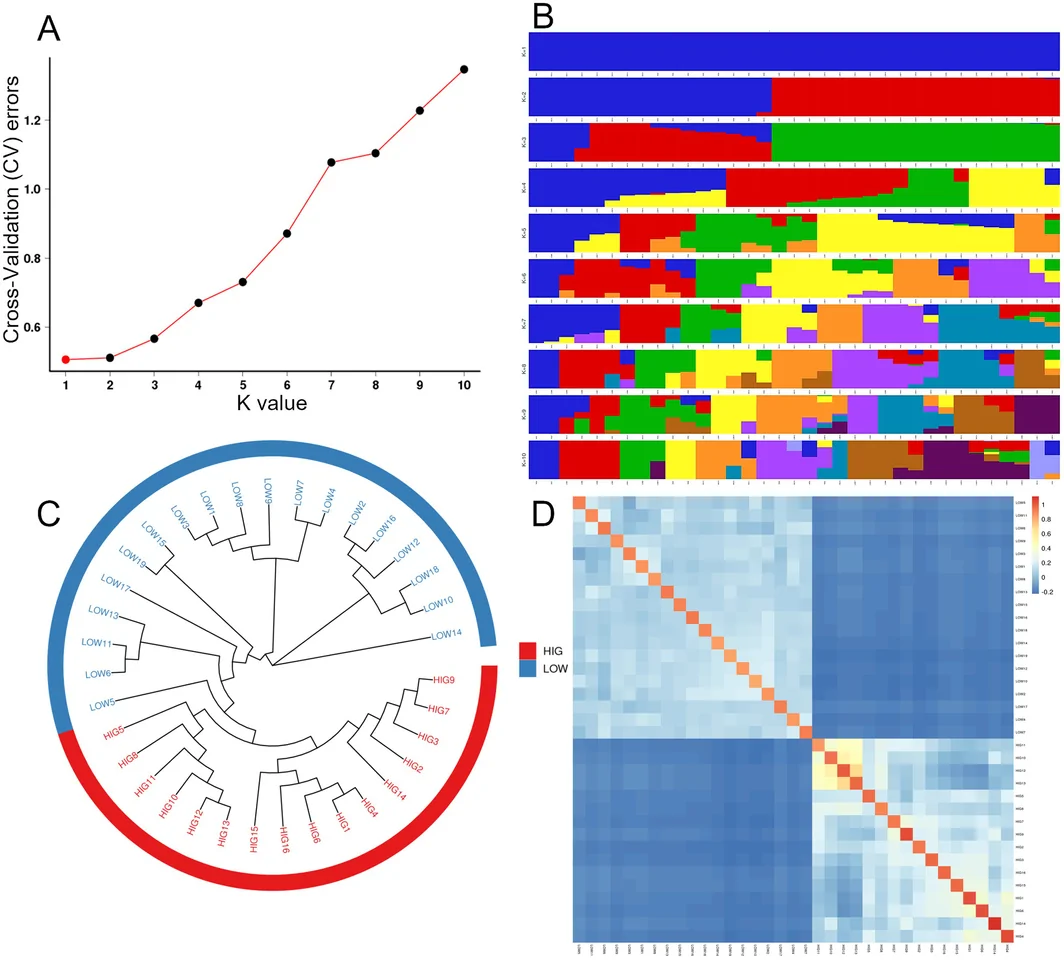
Population genetic structure and phylogenetic relationships of *T. xiaojinensis*. (A) Cross‐validation (CV) error for *K* = 1–10 estimated by ADMIXTURE; the *x*‐axis shows *K* values and the y‐axis shows CV error. The minimum at *K* = 1 indicates the best‐fit number of ancestral populations under this model. (B) Population structure bar plot inferred using 3,903,703 genome‐wide SNPs. Each vertical bar represents one individual (*x*‐axis: Individual ID; *y*‐axis: the proportion of membership in each ancestral cluster). Blue and red segments indicate the ancestry proportion of the two inferred clusters; individuals are sorted by ecotype. (C) Circular neighbor‐joining phylogenetic tree based on genome‐wide SNP data. Outer labels indicate individual IDs; colored branches indicate Low ecotype (blue) and High ecotype (red). (D) Heatmap of pairwise genetic relatedness among 35 individuals calculated by GCTA. The *x*‐ and *y*‐axes list individual IDs grouped by ecotype; the color scale ranges from low (blue) to high (red) relatedness coefficient.

Differentiation between the two ecotypes was quantified as Weir and Cockerham *F*_ST_ in 100‐kb windows stepping by 10 kb. Window values were continuously distributed rather than falling into discrete blocks, giving no separate islands of divergence, with a median of 0.140 and a mean of 0.153 and empirical 95th and 99th percentiles of 0.297 and 0.416 (Figure 4A). 56.3% of windows fell below 0.15, a further 33.9% between 0.15 and 0.25 and 9.9% above 0.25. Nucleotide diversity was close between the ecotypes, with a median of 1.17 × 10^−4^ in the High ecotype and 1.23 × 10^−4^ in the Low ecotype over 30,311 non‐overlapping 100‐kb windows, a consistent but small difference of about 5% that is significant at this sample size (Wilcoxon signed‐rank *p* = 5.3 × 10^−4^, Figure 4B). Against this background the 100‐kb window containing *HSP60A* (Chr04.g05884, 74,500,001–74,600,000 on chromosome 4) stood out. All windows were retained in the genome‐wide scan, but because windows with too few segregating sites give unstable estimates the 99th percentile was also computed after excluding the 16.5% carrying π below 0.5 × 10^−4^ in either ecotype, giving 0.373, and the *HSP60A* window reached *F*_ST_ = 0.391, above this π‐filtered threshold and within the top 1.4% of genome‐wide windows. Nine flanking windows within ±500 kb of the HSP60A start exceeded this value but were all low‐information, and after the same filtering the *HSP60A* window was the most differentiated of the 41 retained in the interval (Figure 4C). Nucleotide diversity within the window was also lower in the High ecotype (0.92 vs. 1.46 × 10^−4^), a 37% reduction confined to the locus against the genome‐wide medians of 1.17 and 1.23 × 10^−4^.

**FIGURE 4:**
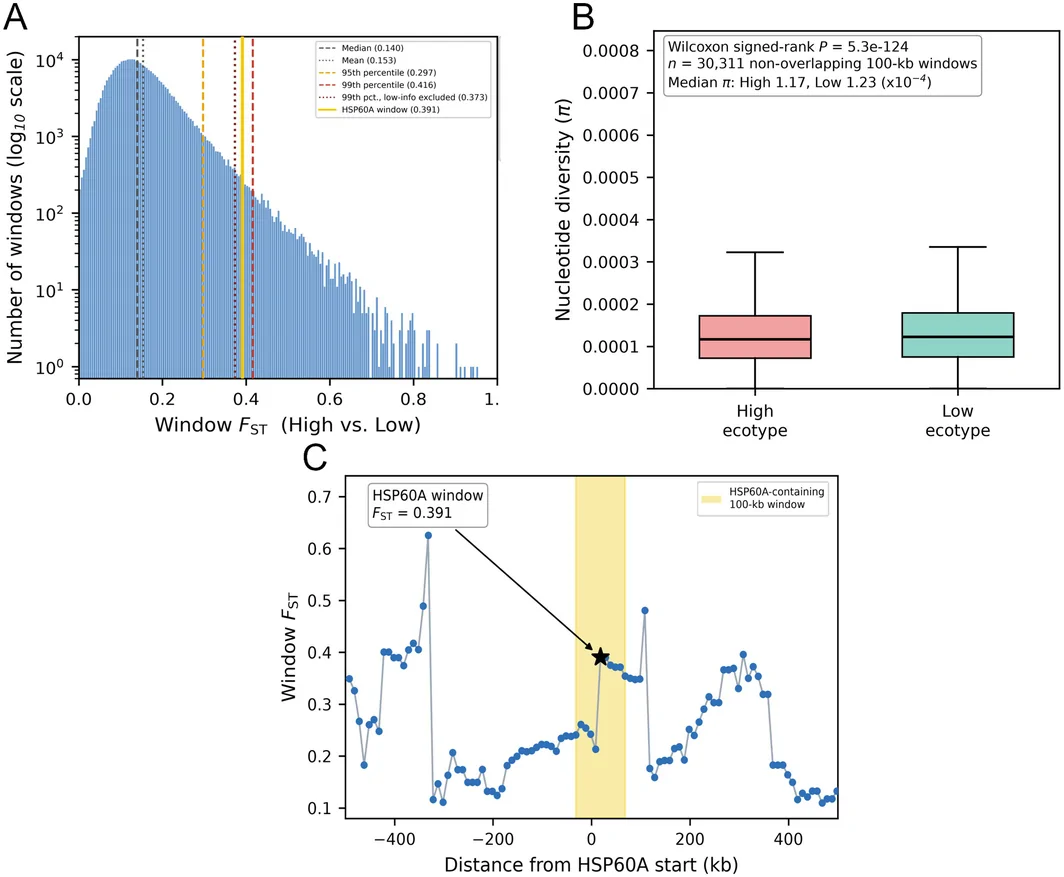
Genome‐wide distribution of window *F*_ST_ and nucleotide diversity, and local differentiation around the *HSP60A* region, in *T. xiaojinensis*. (A) Distribution of Weir & Cockerham *F*_ST_ across 302,630,100‐kb sliding windows (10‐kb step) on the 32 assembled chromosomes. Vertical lines mark the median (0.140), the mean (0.153), the empirical 95th (0.297) and 99th (0.416) percentiles, the 99th percentile after exclusion of low‐information windows (0.373), and the *HSP60A*‐containing window (0.391). The *y*‐axis is on a log10 scale. (B) Nucleotide diversity (π) in the High and Low ecotypes, estimated on 30,311 non‐overlapping 100‐kb windows. Boxes show medians and interquartile ranges (median π: High 1.17, Low 1.23 × 10^−4^); the difference is significant (Wilcoxon signed‐rank test, *p* = 5.3 × 10^−124^). Fliers are shown as gray dots. (C) *F*_ST_ in 100‐kb windows plotted against the distance from the *HSP60A* start site (±500 kb). The shaded band marks the *HSP60A*‐containing window (*F*_ST_ = 0.391); all nine windows in this interval with higher *F*_ST_ are low‐information windows (π < 0.5 × 10^−4^ in either ecotype), so that after the same filtering the *HSP60A* window is the most differentiated of the 41 retained windows.

Plotted along the chromosomes, elevated windows appeared on every one of the 32 rather than clustering in a few, and the per‐chromosome means varied only slightly. To confirm that this landscape is not an artefact of comparing only two ecotype samples, the ecotype labels were permuted among the 35 individuals 999 times and all window *F*_ST_ values were recomputed. The permuted genome‐wide mean *F*_ST_ was −0.0004 (SD 0.008), and windows above the 0.15 and 0.25 thresholds were roughly 900‐fold and 2000‐fold rarer than observed (0.049% vs. 43.7% and 0.005% vs. 9.87%), so every observed statistic, the mean, the fraction above each threshold, the maximum and the value at the *HSP60A* window, lay outside the permutation null (*p* = 0.001). A moving‐block bootstrap over the 10‐kb segments placed the 95% confidence interval of the *HSP60A* window at 0.19–0.81 (Figure 5).

**FIGURE 5:**
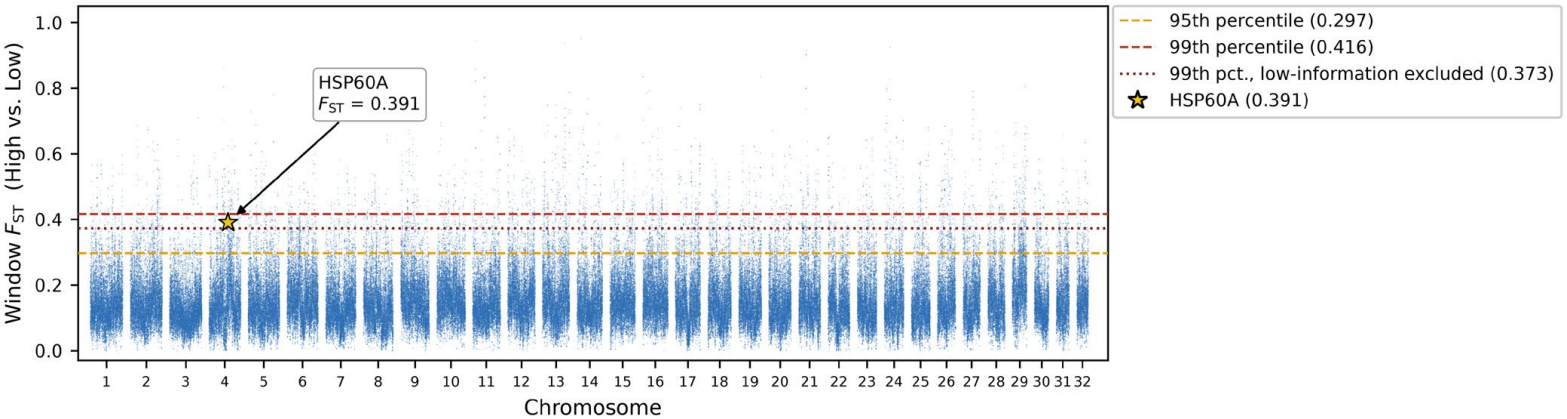
Manhattan plot of *F*_ST_ across the genome. Dashed lines mark the empirical 95th (0.297) and 99th (0.416) percentiles of the genome‐wide window distribution. The window containing *HSP60A* on chromosome 4 (Chr04.g05884) reached 0.391, above the 95th percentile and, once low‐information windows are excluded, above the π‐filtered 99th percentile (0.373). A moving‐block bootstrap over the 10‐kb segments (2000 replicates) placed the 95% confidence interval of this window at 0.19–0.81, and it exceeded all 999 ecotype‐label permutations (*p* = 0.001).

### 3.4 Identification and Orthogonal Validation of the *HSP60A* Candidate Variant

Read alignment across the 35 individuals recovered a G → T transversion in exon 7 of *HSP60A* (c.1132G>T, p.Glu378*) that changes codon 378 from GAG to TAG, truncating the protein from 571 to 377 residues and removing the C‐terminal part of the discontinuous equatorial domain while leaving the ATP‐binding N‐terminal segment intact (Figure 6A,B). The derived allele appeared in twelve of the 19 Low‐ecotype individuals (0.63) and was not detected in any of the 14 High‐ecotype individuals with reads covering the position, the remaining two having no reads at the site. Parsimony against the outgroup assigns glutamate as the ancestral state, conserved in all 11 lepidopteran *HSP60* orthologues examined and replaced only in the vertebrate orthologue and two more distant chaperonin‐60 relatives, while no sequence carries a stop there, so the polarity rests on amino‐acid conservation rather than codon‐level reconstruction (Figure 6C).

**FIGURE 6:**
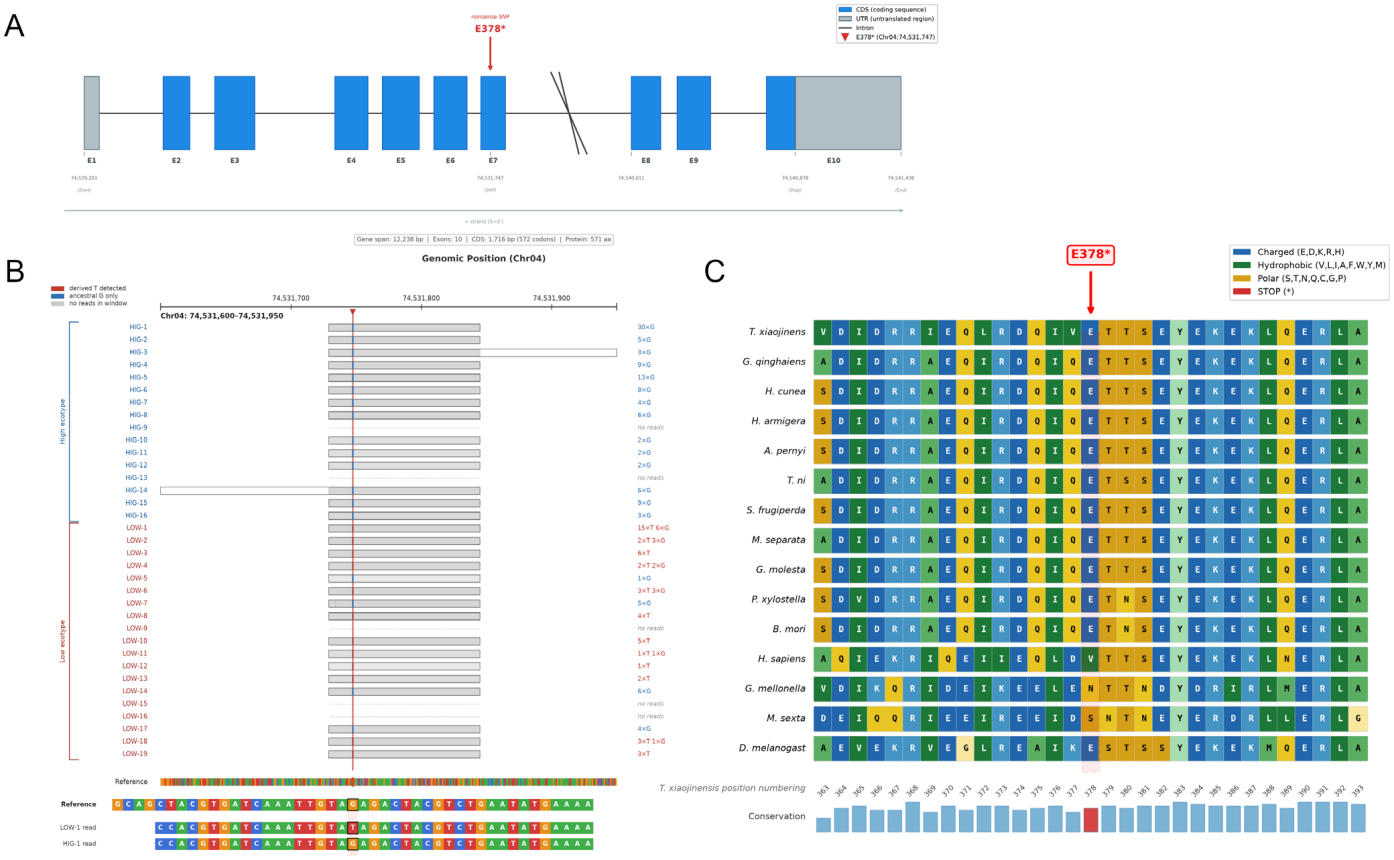
Identification of the candidate variant at the *HSP60A* locus in *T. xiaojinensis*. (A) Read alignment at the *HSP60A* locus (Chr04:74,531,600–74,531,950). Each row is one of the 19 Low‐ and 16 High‐elevation individuals; gray bars are reads, and the bases observed at the variant position are counted on the right. The derived G → T variant (c.1132G>T, p.Glu378*) is marked by the red line. Individuals with no reads in the window are labelled, and among High‐elevation individuals with coverage, the ancestral G is the only allele detected. (B) Gene structure of *HSP60A* (10 exons), with the c.1132G>T variant in exon 7 creating a premature stop codon (p.Glu378*) that truncates the protein from 571 to 377 residues and removes the C‐terminal segment of the discontinuous equatorial domain. (C) Multi‐species protein alignment around position 378. Glu378 is invariant among the 11 lepidopteran *HSP60* orthologues examined, and no aligned sequence carries a stop at this position.

An orthogonal PCR assay recovered the derived T allele in four of the five Low‐elevation individuals and in none of the five High‐elevation individuals (Figure 7), matching the SLAF‐based allele frequency and its restriction to the Low ecotype. With 0 of 10 High‐elevation chromosome copies carrying the derived allele, the one‐sided 95% upper bound on its frequency is 26%, which excludes a substantial High‐elevation frequency but not rarity. The two assays therefore agree, and the allelic series at *HSP60A* is supported by both genome‐wide genotyping and locus‐specific sequencing.

**FIGURE 7:**
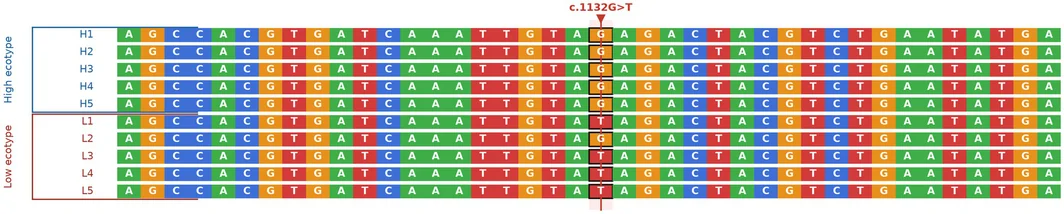
Locus‐specific validation of the *HSP60A* candidate variant by PCR amplification and Sanger sequencing. Each row is one individual and each cell one nucleotide, showing the 40‐bp segment surrounding the variant. The boxed column and the red line mark the variant (c.1132G>T, p.Glu378*). The derived T allele was present in four of the five Low‐elevation individuals and in none of the five High‐elevation individuals, in which only the ancestral G was detected.

### 3.5 Thermal Stress‐Induced Transcriptional Divergence Between High and Low Ecotypes

Following the identification of a highly differentiated *HSP60A* variant between the ecotypes, transcriptional responses to thermal stress (8°C, 12°C, and 16°C) were profiled to investigate potential regulatory divergence. Expression quantification and inter‐sample correlation analysis confirmed high data quality (pairwise Pearson *r* > 0.95 within replicates), revealing distinct global expression patterns between ecotypes across temperature treatments (Figure 8). Comparative heatmaps of the top 1000 most variable genes revealed distinct expression profiles between High and Low ecotypes at all three temperatures (Figure 9). Pairwise comparisons across temperatures within each ecotype revealed that gene expression patterns remained largely stable across the 8°C–16°C range within each ecotype, indicating transcriptional homeostasis (Figure 10A–C). In contrast, the between‐ecotype comparison at 16°C amounted to a modest global shift, the High ecotype carrying a slightly higher mean expression across all genes than the Low ecotype (mean log_10_ TPM 1.03 vs. 0.97, Mann–Whitney *U* test *p* < 0.01, Figure 10D). This reflects a difference in baseline transcription between the ecotypes at the whole‐transcriptome level.

**FIGURE 8:**
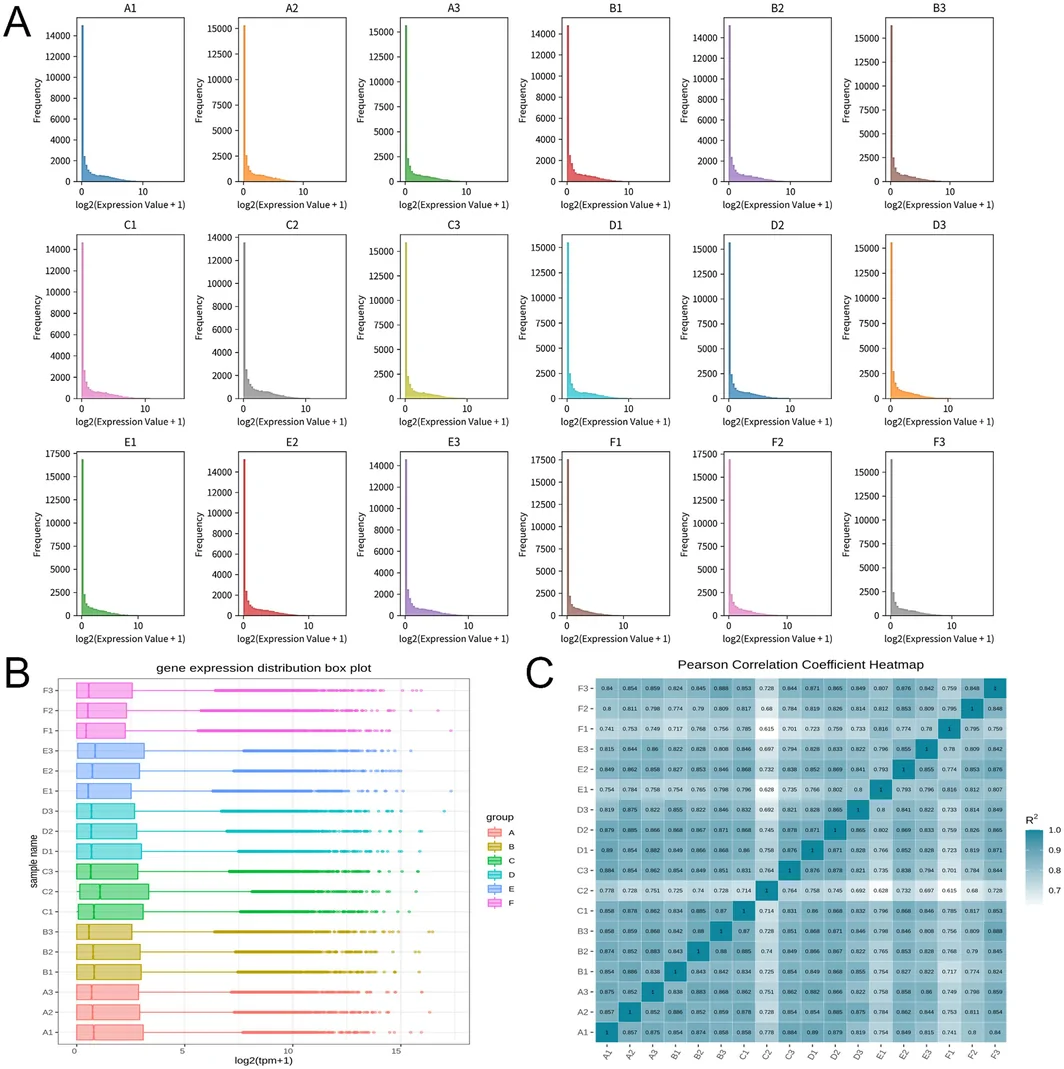
Sample quality control and expression quantification. (A) Histogram of expression level distribution across samples. The *x*‐axis shows log_10_ (TPM + 1) expression values; the y‐axis shows the number of genes. (B) Box plots of the expression distribution of each of the 18 samples (*x*‐axis: Log_2_ (TPM + 1); *y*‐axis: Sample ID); samples are colored by experimental group (two ecotypes × three temperatures, 18 libraries in total). (C) Pearson correlation heatmap of all pairwise sample correlations. The *x*‐ and *y*‐axes list all 18 sample IDs; the color scale represents the Pearson correlation coefficient (*r*), from 0.7 (light) to 1.0 (dark). Within‐replicate correlations are shown in the diagonal blocks.

**FIGURE 9:**
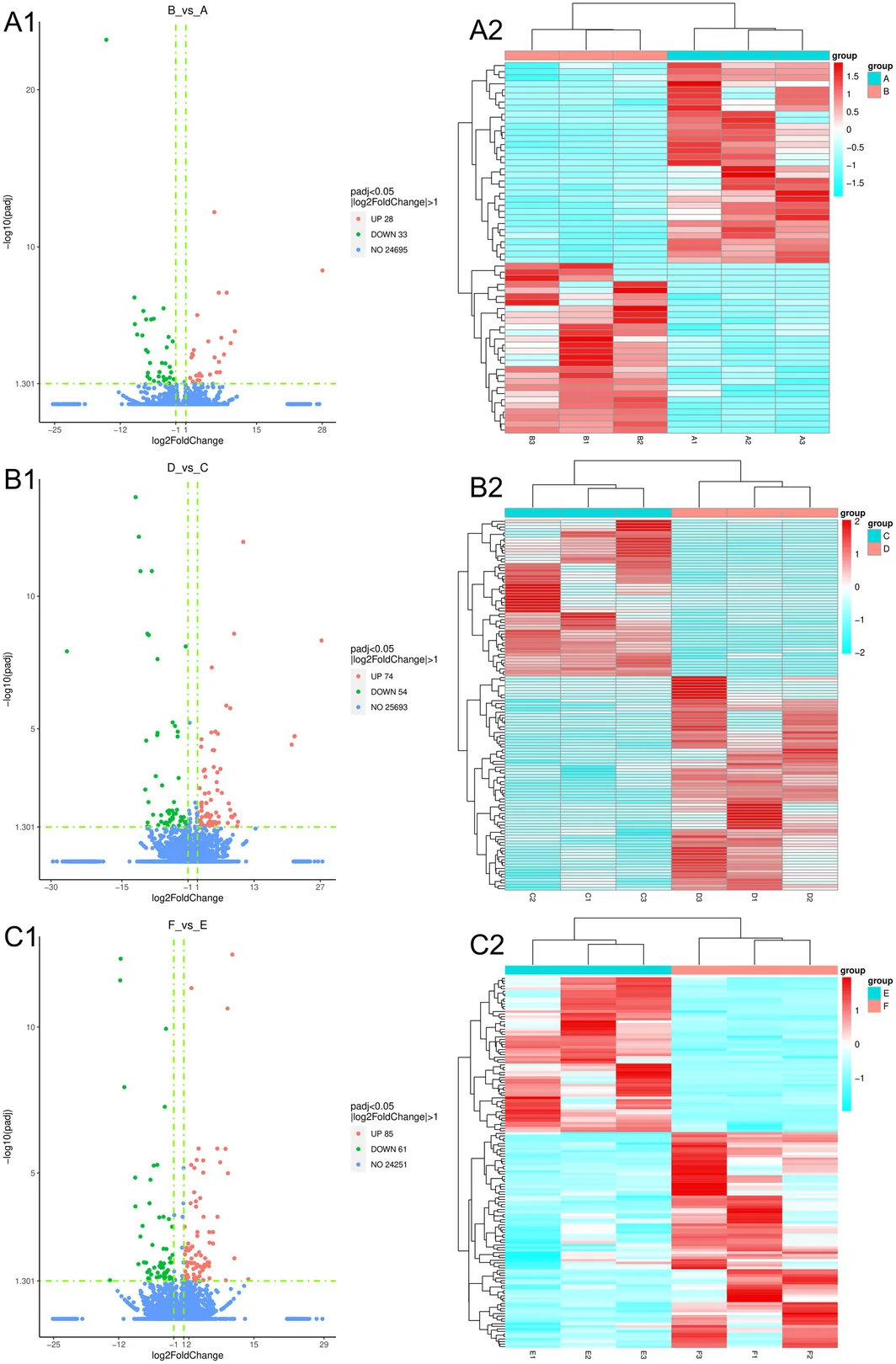
Differential expression between High and Low ecotypes under different temperature treatments. (A1) Volcano plot; (A2) Heatmap—High ecotype versus Low ecotype at 8°C. (B1) Volcano plot; (B2) Heatmap—High ecotype versus Low ecotype at 12°C. (C1) Volcano plot; (C2) Heatmap—High ecotype versus Low ecotype at 16°C. In volcano plots, the *x*‐axis is log_2_ fold change and the *y*‐axis is –log_10_ (adjusted *p* value); dashed lines mark the thresholds (adjusted *p* < 0.05 and |log_2_ fold change| > 1), and points are colored by regulation direction (red, up‐regulated; green, down‐regulated; blue, not significant). In the heatmaps, rows are genes and columns are individual samples, with sample groups indicated by the color bar above each panel; the dendrograms show hierarchical clustering of genes and samples, and the color scale represents scaled expression (cyan, low; red, high). The three heatmaps share the same gene set and the same row order, and the sample columns are ordered identically in A2, B2 and C2, so that differences among panels reflect expression rather than reordering.

**FIGURE 10:**
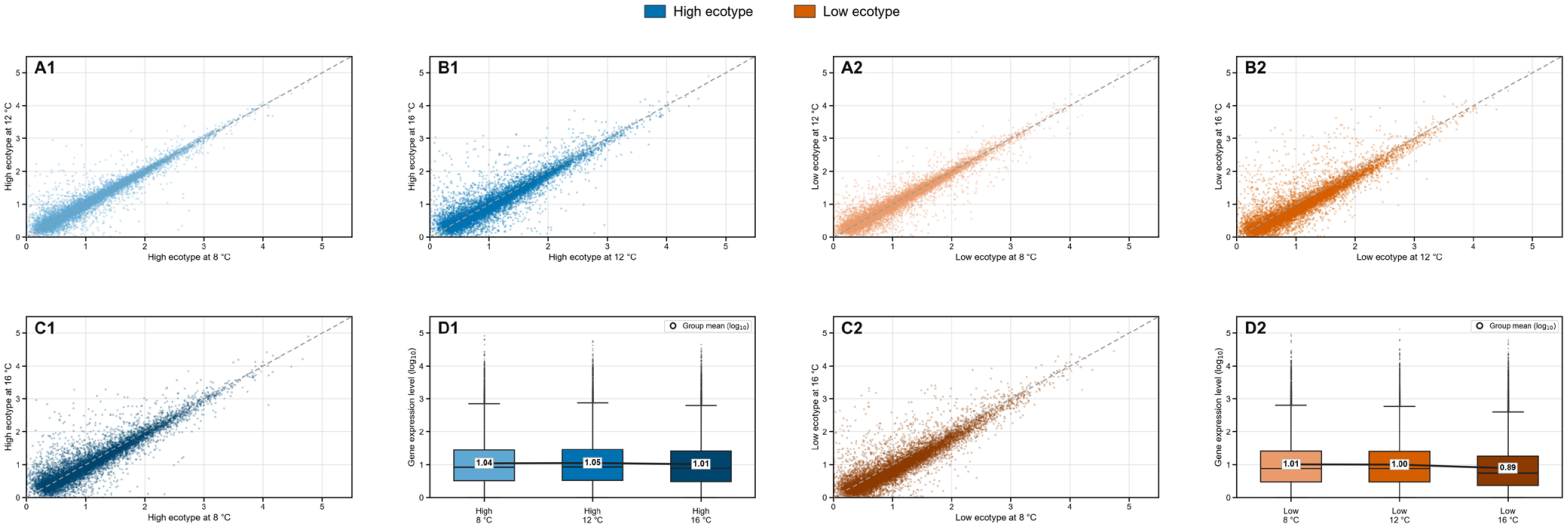
Gene expression patterns within ecotypes across temperature treatments. (A1) High ecotype at 8°C versus 12°C. (A2) Low ecotype at 8°C versus 12°C. (B1) High ecotype at 12°C versus 16°C. (B2) Low ecotype at 12°C versus 16°C. (C1) High ecotype at 8°C versus 16°C. (C2) Low ecotype at 8°C versus 16°C. (D1) Box plot of log_10_ (TPM + 1) for the High ecotype across 8°C, 12°C, and 16°C (*x*‐axis: Temperature group; *y*‐axis: Log_10_ (TPM + 1)). (D2) Same layout for the Low ecotype.

### 3.6 Two Chaperone Systems With Divergent Thermal Responses

A signed hybrid WGCNA network of the 11,046 expressed genes reached its best scale‐free fit at *β* = 6 (*R* ^2^ = 0.75, mean connectivity 296), a power short of the conventional *R* ^2^ ≥ 0.85 (Table S3), and resolved into 35 modules carrying 10,423 genes with 623 left unassigned (Figure 11A–C). Connectivity was strongly asymmetric, the largest turquoise module holding a median kWithin of 131.4 against 4.8 genome‐wide (Figure 11D).

**FIGURE 11:**
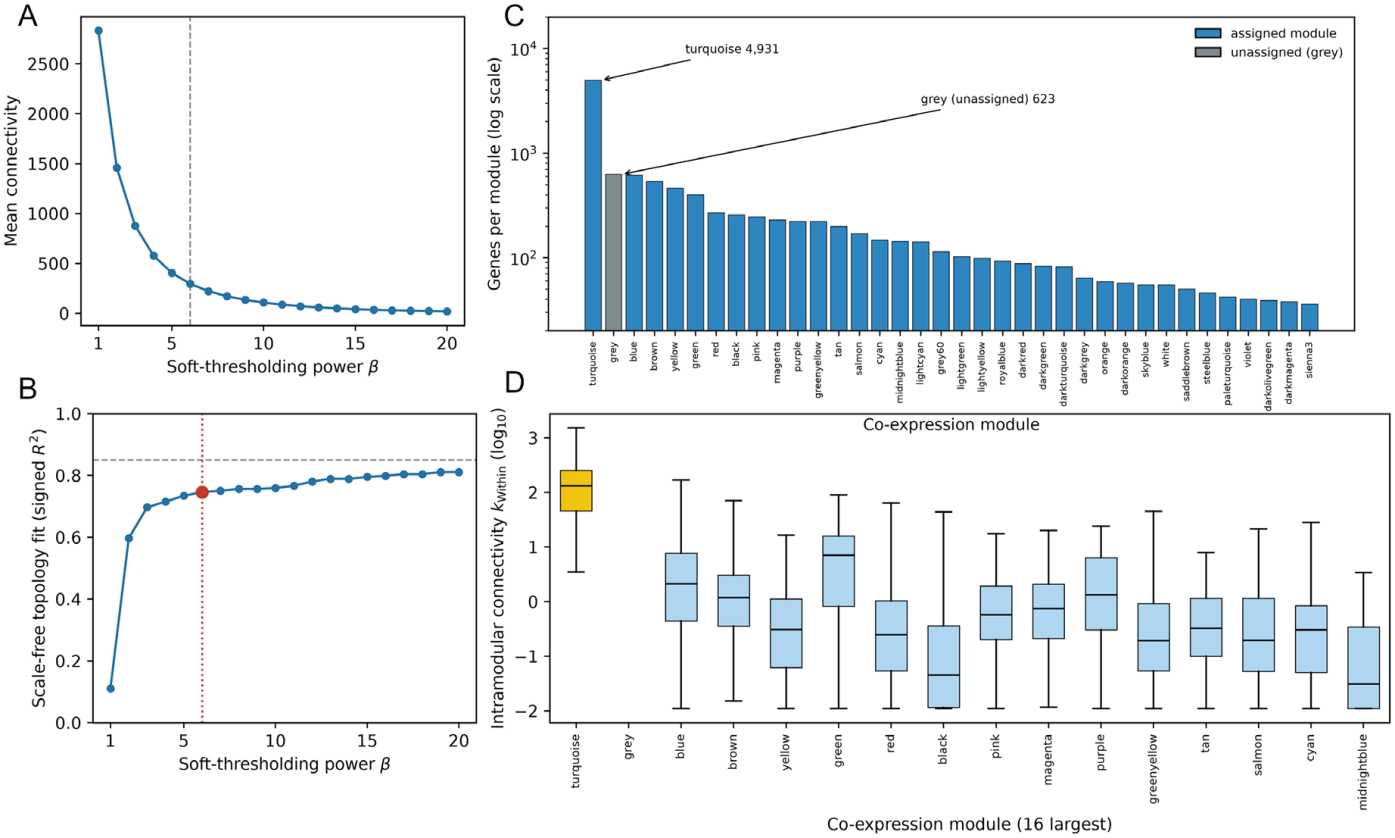
Weighted gene co‐expression network analysis. (A) Mean connectivity as a function of the soft‐thresholding power *β*. (B) Signed scale‐free topology model fit as a function of *β*; the dashed line marks *R* ^2^ = 0.85 (not reached by this data set) and the red point marks *β* = 6, the value used. (C) Number of genes per co‐expression module (35 modules; the gray bar shows the 623 genes not assigned to any module). (D) Intramodular connectivity (kWithin) of the 16 largest modules (log_10_ scale); boxes show medians and interquartile ranges. All panels are based on the 11,046 expressed genes across 18 samples. Module number and individual module assignments are sensitive to the expression scale; the family‐level contrasts reported in the text were verified under three normalisations.

The module eigengenes divided the transcriptome by thermal behavior, turquoise falling as assay temperature rose (*r* = −0.567, *p* = 0.014) while the black module and the unassigned genes rose with it (*r* = +0.656, *p* = 0.0031 and *r* = +0.627, *p* = 0.0054, Figure 12A). Twelve of the 21 small heat‐shock proteins fell into these temperature‐rising sets, against 1.7 expected, an enrichment of about sevenfold (hypergeometric *p* = 9.1 × 10^−9^, Figure 12B), and they were strongly induced from 8°C to 16°C, a median of +1.21 log_2_ units against −0.25 for the classical chaperones (Figure 12C). This inducible versus non‐inducible split held on raw counts (Mann–Whitney *p* = 7.9 × 10^−6^), on the canonical classical subset (*p* = 6.6 × 10^−5^) and after DESeq2 size‐factor correction (*p* = 7.3 × 10^−6^), and the two systems were anticorrelated overall (biweight midcorrelation −0.17). *HSP60A*'s co‐expression neighborhood places it in the non‐inducible classical arm, with positive biweight midcorrelation to the classical chaperone partners and negative to the chromosome‐12 small‐HSP cluster (Figure 12D).

**FIGURE 12:**
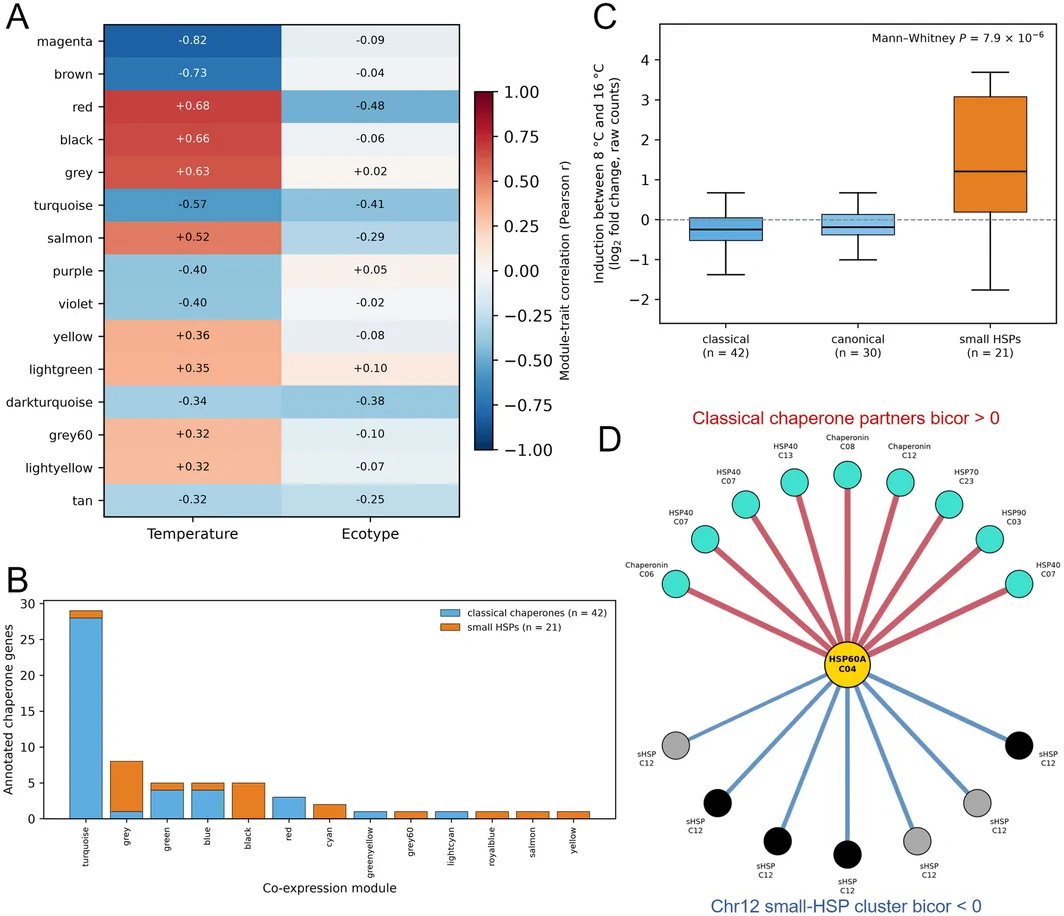
Co‐expression structure and thermal behavior of the chaperone complement. (A) Pearson correlation between module eigengenes and assay temperature and ecotype, shown for the 15 largest of the 35 modules. Cell fill gives the correlation coefficient, red for positive and blue for negative. The ecotype was coded High = 0 and Low = 1, so a positive value in the Ecotype column means a higher eigengene level in the Low ecotype. The four modules with the strongest positive correlation with temperature (black, gray, red and salmon, all *r* ≥ 0.5) and the three with the strongest negative correlation (magenta, brown and turquoise) separate the two chaperone systems. (B) Numbers of annotated classical chaperone and small *HSP* genes assigned to each co‐expression module, with bar fill indicating gene family (classical chaperones in blue, small *HSPs* in orange) and module names given on the *x*‐axis. (C) Induction between 8°C and 16°C for the classical chaperone set (*n* = 42), its canonical subset (*n* = 30) and the small *HSP* set (*n* = 21), on the raw‐count scale; boxes show medians and interquartile ranges. (D) Co‐expression neighborhood of *HSP60A*, with red edges marking positive biweight midcorrelation to classical chaperone partners and blue edges marking negative biweight midcorrelation to the chromosome‐12 small‐HSP cluster; the star layout displays pairwise correlations with *HSP60A* and does not imply intramodular hub status, in which *HSP60A* is peripheral. Node fill indicates the WGCNA module of each partner (black = black module, gray = unassigned gray module, turquoise = turquoise module) and the two‐line label gives gene family and chromosome; edges are drawn for the top classical and top Chr12 small‐HSP partners by |bicor| at |bicor| ≥ 0.35.

That placement is robust and carries through to the ecotypes and to survival. The classical‐versus‐small‐HSP biweight midcorrelation contrast for *HSP60A* (median + 0.56 vs. −0.27, *p* = 3.5 × 10^−9^) survived every normalisation tested, +0.40 versus −0.17 under log_2_(CPM + 1) and +0.07 versus −0.08 under variance‐stabilized counts (Figure 13A), and within its module *HSP60A* stayed peripheral, in the lowest‐connectivity tier rather than among the hubs. *HSP60A* was expressed at lower levels in the Low than in the High ecotype across all three temperatures, a contrast significant under log_2_(TPM + 1) (two‐way ANOVA, *p* = 0.020) but not under log_2_(CPM + 1) (*p* = 0.080) or variance‐stabilized counts (*p* = 0.642, Figure 13B, Table 2). The two arms separated the ecotypes as well, the small *HSPs* running 3.8‐fold higher in the Low than the High ecotype at 16°C (+1.94 log_2_, *p* = 5.5 × 10^−7^) and inducing 2.6‐fold more strongly there (+1.40 log_2_, *p* = 7.5 × 10^−3^), both reproduced under the alternative scales, while the classical arm showed no ecotype difference after background correction (Figure 13C). In the mortality assays the Low ecotype survived better than the High ecotype at 16°C (*p* = 0.0342, Figure 13D).

**FIGURE 13:**
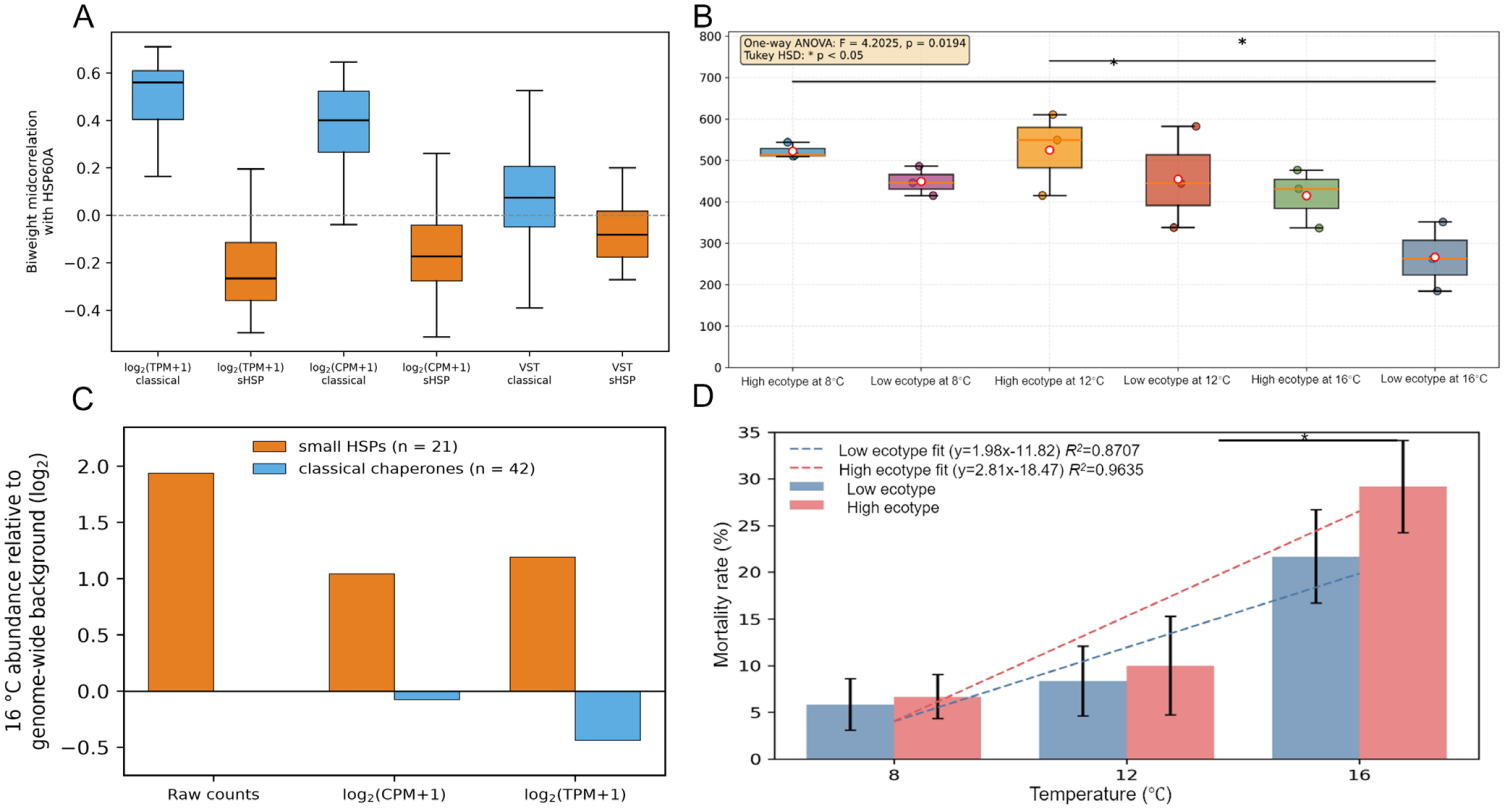
*HSP60A* co‐expression contrast, expression and larval mortality. (A) Distribution of the biweight midcorrelation between *HSP60A* and the classical chaperone genes (excluding *HSP60A* itself; *n* = 41) and between HSP60A and the small HSP genes (*n* = 21), computed under three normalisations (log_2_(TPM + 1), log_2_(CPM + 1) and variance‐stabilized counts); boxes show medians and interquartile ranges. (B) *HSP60A* expression across ecotype × temperature groups (*x*‐axis: Group label; *y*‐axis: Log_2_(TPM + 1)); each group comprises three biological replicates. (C) Abundance at 16°C of the small *HSP* and classical chaperone sets relative to the genome‐wide background shift, under three normalisations. Each group comprises three biological replicates, giving 18 libraries in total, and the boxes show the distribution across libraries within each group. (D) Mortality rates of High and Low ecotypes at 8°C, 12°C, and 16°C (*x*‐axis: Temperature; *y*‐axis: Mortality %, mean ± SE). Each data point represents the mean of 3 biological replicates (*n* = 30 larvae per replicate); error bars = standard error of the mean. Statistical significance between ecotypes at each temperature was evaluated by unpaired *t*‐test (*: *p* < 0.05; ns: *p* ≥ 0.05).

**TABLE 2:** Sensitivity of the reported *HSP* contrasts to the expression scale.

Contrast	log_2_(TPM + 1)	log_2_(CPM + 1)	VST/negative‐binomial
*HSP60A* ecotype effect (two‐way ANOVA P)	0.020	0.080	0.642/0.803
*HSP60A* Low/High fold change	0.773	0.842	0.961/0.978
Classical versus *sHSP* induction, 8 → 16°C (P)	1.3 × 10^−5^	3.4 × 10^−5^	Raw counts 7.9 × 10^−6^
Two‐arm ecotype interaction (P)	3.2 × 10^−3^	5.1 × 10^−4^	Raw counts 7.5 × 10^−3^
16°C *sHSP* abundance over background (log_2_)	+1.19	+1.05	Raw counts +1.94
*HSP60A* excess over background (log_2_)	−0.31	−0.10	Raw counts −0.03

## 4 Discussion

### 4.1 Early‐Stage Ecotypic Divergence Under Ongoing Gene Flow

The genome of *T. xiaojinensis* is weakly structured overall, yet it partitions by elevation at *K* = 2 and carries elevated differentiation on every chromosome. The pattern is what theory predicts for divergence that started recently and has proceeded without a barrier to gene flow. Read against theory, the pattern is what gene flow and divergent selection leave behind when separation is recent. Migration homogenises neutral and weakly selected loci, holding the genome‐wide background low, while loci whose fitness tracks the elevational environment resist that homogenization and rise above it (Via 2012; Feder et al. 2012; Rodríguez‐Rodríguez et al. 2020). Selection at that locus must outweigh the migration reaching it. With only about one to two migrants per generation under an island‐model approximation (Nm ≈ 1.4 from the genome‐wide mean *F*_ST_), the per‐generation migration rate is small for any realistic population size, so even moderate selection suffices and differentiation persists at ecologically relevant loci without any barrier to exchange (Shaner et al. 2015). The elevated windows form a continuum rather than discrete blocks. A continuum of this shape is what both polygenic clinal selection and neutral migration‐drift equilibrium can produce, so on its own it neither demonstrates islands nor rules out drift (Agashe and Bolnick 2010), which is precisely why we read the pattern at the level of individual candidate loci rather than as a genome‐wide island architecture. A two‐sample design compels this reading in any case, since a single pair of samples cannot separate stochastic from selection‐driven outliers (Via 2012).

The mitochondrial data point to the same conclusion, that the split is recent. The two ecotype samples form reciprocally monophyletic COI clusters, so each carries a distinct mitochondrial lineage, which is compatible with continued nuclear gene flow because the mitochondrial genome sorts faster. Yet their K2P distance of 3.67% lies well below the interspecific distances computed among congeneric Thitarodes (mean 9.98%, and 13.66% among congeners generally, from published COI sequences, Dai et al. 2019, Wang et al. 2019). The shallow coalescence between the two mtDNA clades therefore marks a split young enough that the ecotypes remain intraspecific variants rather than cryptic species. That mtDNA has already sorted into reciprocal clades while the nuclear genome stays weakly structured is itself the signature of very recent divergence, the small mitochondrial effective size sorting lineages far faster than the nuclear genome. Some Low‐ecotype individuals carry High‐ecotype ancestry, which is what one expects if the split is recent and gene flow has continued. Allopatric separation with secondary contact cannot be excluded, because it can mimic these ADMIXTURE patterns. The continuous elevational gradient, however, is a standing driver of local adaptation, and it supplies the selective contrast the parapatric reading requires (Tonin et al. 2020).

### 4.2 *HSP60A* as a Candidate Locus Under Divergent Selection

*HSP60A* is the most differentiated window that survives the low‐information filter in an otherwise modest landscape, reaching *F*_ST_ = 0.391 and lying outside the permutation null, yet an outlier is not by itself evidence of selection and with two ecotype samples a fraction of windows must differ by chance alone (Via 2012). We therefore treat it as a candidate rather than a confirmed target. The allele pattern is asymmetric, the ancestral allele fixed among High‐ecotype individuals with coverage while the derived nonsense mutation is confined to the Low ecotype, and nucleotide diversity is reduced in the High ecotype at the same locus. These features admit two selective readings: a sweep fixing the ancestral haplotype in the High ecotype or directional selection favouring the derived allele in the Low ecotype, but linked selection and drift remain live alternatives that the present data cannot separate (Feder and Nosil 2010; Via et al. 2012).

A regulatory or promoter variant in linkage with the coding site could produce both the allele‐frequency contrast and the expression difference at 16°C, and two ecotype samples cannot break that linkage statistically. What the coding position offers is a direct route to a functional consequence that a purely regulatory explanation would leave unexplained, reducing the nonsense mutation to a passive passenger. As the only outlier annotated to the heat‐stress response pathway, *HSP60A* is the most plausible candidate for selection‐driven differentiation here (Kontopoulos et al. 2020), and divergent selection can hold such a locus apart despite ongoing gene flow (Edelaar et al. 2023), but demographic and linked‐selection explanations therefore remain open.

Allele polarity rests on explicit evidence and matters even under neutrality. Parsimony against the outgroup assigns glutamate as ancestral, a residue invariant across the lepidopteran orthologues examined with no aligned sequence carrying a stop, so the stop is the derived state and purifying selection on this residue is the parsimonious reading within Lepidoptera. The two states are not functionally equivalent. The derived allele truncates the chaperonin and removes the C‐terminal segment of the discontinuous equatorial domain that contributes to oligomer assembly, while the ancestral allele encodes the full‐length protein (Braig et al. 1994; Horwich et al. 2007), and a premature termination codon of this kind also renders the transcript liable to nonsense‐mediated decay (Lykke‐Andersen and Jensen 2015). Polarity is therefore what identifies which ecotype carries a loss of function and gives direction to the frequency contrast, even if the allele's spread were neutral. Whether the derived allele was itself favoured by positive selection remains open pending haplotype‐based tests and coalescent simulations.

### 4.3 *HSP60A* in the Non‐Inducible Chaperone System Links a Coding Variant to the Thermal Phenotype

The co‐expression network shows where the coding variant sits in the chaperone system, and therefore what kind of effect it can have. The chaperone complement resolves into two systems with opposed thermal behavior, a strongly heat‐inducible small‐HSP arm and a non‐inducible classical arm, and *HSP60A*, a chaperonin, co‐expresses with the classical arm rather than the inducible one, a placement that holds across normalisations (Horwich et al. 2007). The divergence at *HSP60A* therefore does not run through the acute heat‐shock response, and its transcript difference between ecotypes is a baseline reduction rather than a shift in inducibility.

The coding change supplies a direct route to that baseline shift. The E378* nonsense mutation removes about a third of the protein, deleting the C‐terminal segment of the discontinuous equatorial domain and the adjacent intermediate region that together form the oligomerization interface, while sparing the ATP‐binding N‐terminal segment (Braig et al. 1994, Horwich et al. 2007), and a premature stop of this kind is expected to lower steady‐state transcript through nonsense‐mediated decay (Lykke‐Andersen and Jensen 2015). Truncation and transcript decay together leave the Low ecotype operating with reduced *HSP60A* capacity. Because the variant sits in the coding sequence, it can account for the genotype‐transcript association on its own, though a linked regulatory variant cannot be ruled out with the present data.

The ecotype difference in the transcriptome instead sits in the inducible arm, where the small *HSPs* are more abundant and more strongly induced in the Low ecotype at 16°C while the classical arm shows none after background correction. The two ecotypes therefore diverge on two axes at once, a constitutively reduced chaperonin and a strengthened inducible defence, and it is the latter that tracks the lower mortality of the Low ecotype at 16°C. Less baseline chaperonin paired with a keener inducible response is the signature of an elevated heat‐shock threshold, plausibly favoured because maintaining chaperonin activity is costly, and it echoes the reduced‐expression‐with‐better‐tolerance pattern seen for *Hsp70* in Drosophila buzzatii (Moseley 1997; Sørensen et al. 2001). That the two ecotypes were reared in parallel under identical conditions and assayed at the same temperatures makes this a genetic contrast rather than a plastic one, which common‐garden rearing would otherwise erase. Whether *HSP60A* is the direct target of selection is a question for functional tests.

### 4.4 Conservation Implications and Future Directions

The ecotypes differ at *HSP60A* and in their inducible defence while gene flow continues, so they are best treated as components of a single adaptive continuum rather than separate management units. With *O. sinensis* declining under warming, three readings follow. Locally adapted low‐expression variants may be swamped as high‐elevation habitats warm, or the same gene flow may carry alleles now common in the Low ecotype uphill and raise fitness there. A third possibility follows from the moth's own population dynamics, because *O. sinensis* is the principal source of larval mortality, so a decline in fungal abundance could relax that mortality and raise moth density even where thermal conditions deteriorate. Whether this translates into more hosts for harvest depends on fungal density, which we did not measure. Which reading prevails turns on the fitness effect of E378*, not yet measured, so both stay open and the robust posture is to preserve the full range of thermal‐tolerance variation, including both allele classes. Testing that fitness effect, confirming the truncation and its decay and compensation, and extending from one locus to genome‐wide and long‐term field monitoring will settle it.

## 5 Conclusion

Population genomics, transcriptomics and survival assays together indicate that the High and Low ecotypes of *T. xiaojinensis* are diverging along the elevational gradient while gene flow continues. Against a genome‐wide background in which elevated windows occur on every chromosome rather than in a few discrete regions, *HSP60A* is the clearest single‐locus signal, a coding nonsense mutation (p.Glu378*) that exceeds the 99th percentile after low‐information windows are excluded and lies within the top 1.4% of genome‐wide windows, is confirmed by independent sequencing, and is carried mainly by the Low ecotype. Its meaning lies in where it sits in the regulatory landscape. Co‐expression analysis separates the chaperome into a heat‐inducible small‐HSP arm and a non‐inducible classical arm, places *HSP60A* firmly in the latter, and shows the Low ecotype running this constitutive chaperonin lower while mounting a stronger inducible small‐HSP response and surviving warmth better. A coding change in a housekeeping chaperonin and a re‐tuned inducible defence thus appear to act together, and an elevated heat‐shock threshold in the Low ecotype is a working model rather than a proven mechanism, since the expression contrast is scale‐dependent and the fitness cost of the truncation is unmeasured. From a conservation perspective, the intraspecific variation harbored by these ecotypes constitutes the evolutionary raw material enabling the species to cope with plateau warming. Retention of both morphotypes and allelic classes represents a more prudent strategy, as the fitness consequences of this truncation variant remain uncharacterized.

## Author Contributions

**Qiuyang Wei:** conceptualization (equal), formal analysis (equal), investigation (equal), methodology (equal), visualization (equal), writing – original draft (lead), writing – review and editing (equal). **Yuanchuan He:** formal analysis (equal), investigation (equal), methodology (equal), visualization (equal), writing – original draft (equal), writing – review and editing (equal). **Shuai Pang:** formal analysis (supporting), methodology (supporting), resources (supporting). **Shijiang Chen:** conceptualization (lead), funding acquisition (lead), project administration (lead), resources (lead), supervision (lead), writing – review and editing (lead).

## Funding

National Natural Science Foundation of China (Grant No. U25A20162). The Science and Technology Research Program of Chongqing Municipal Education Commission (Grant No. KJQN202515101). Chongqing Science and Technology Innovation Leading Talent Support Program (2022) (Grant No. CQYC20220301627). The Fundamental Scientific Research Funds Project of Chongqing Municipal Science and Technology Bureau (Grant No. CSTB2025jxjl‐jbkyX0017).

## Conflicts of Interest

The authors declare no conflicts of interest.

## Supporting information


**Table S1:** Protein sequences used for the conservation analysis at position 378 of *Thitarodes xiaojinensis HSP60A*. The set comprises 11 lepidopteran *HSP60* orthologues spanning seven families (the *T. xiaojinensis* reference plus ten additional species), the vertebrate orthologue *Homo sapiens* *HSPD1* (P10809.2), and three more distantly related chaperonin‐60 family members. Positions follow the *T. xiaojinensis* HSP60A numbering; the equivalent position and residue are given for each sequence. Equivalent positions were assigned from the alignment using the conserved YEKEK motif of the lepidopteran orthologues as the anchor.
**Table S2:** The chaperone complement of *T. xiaojinensis*: family assignment, module membership and co‐expression with HSP60A.
**Table S3:** Soft‐threshold selection for scale‐free network construction (signed hybrid adjacency, 11,046 genes × 18 samples). Values are the signed scale‐free topology fit *R* ^2^ and the mean connectivity computed by WGCNA. No tested power met the conventional *R* ^2^ ≥ 0.85 criterion; the fit reached 0.81 only at *β* = 20, by which point mean connectivity had fallen to 19.8 and module sizes had contracted sharply. *β* = 6 (row highlighted) was therefore used for network construction.

## Data Availability

All raw sequence data generated in this study are deposited in the Genome Sequence Archive (GSA) at the National Genomics Data Center (NGDC, China National Center for Bioinformation) under accession numbers CRA038233 (SLAF genomic data for elevational adaptation) and CRA038310 (temperature‐responsive transcriptome). During peer review, the editors and the reviewers can access them through the shared links https://ngdc.cncb.ac.cn/gsa/s/Bu9lE07Y (CRA038233) and https://ngdc.cncb.ac.cn/gsa/s/QXkmP523 (CRA038310).
