# Supplementary material for: Elevational Adaptation in the Moth Thitarodes xiaojinensis: HSP60A‐Mediated Thermal Tolerance Evolution Under Gene Flow
Source: Ecol Evol. 2026 Nov 9;16(11):e74635. doi: 10.1002/ece3.74635 (PMC13746093; doi:10.1002/ece3.74635)
Supplement: Supplementary file 1 [file ECE3-16-e74635-s001.docx]

**Appendix**

The following supplementary materials are provided to enhance the understanding of this study.

Table S1. Protein sequences used for the conservation analysis at position 378 of Thitarodes xiaojinensis *HSP60A*. The set comprises 11 lepidopteran *HSP60* orthologues spanning seven families (the T. xiaojinensis reference plus ten additional species), the vertebrate orthologue Homo sapiens *HSPD1* (P10809.2), and three more distantly related chaperonin-60 family members. Positions follow the T. xiaojinensis HSP60A numbering; the equivalent position and residue are given for each sequence. Equivalent positions were assigned from the alignment using the conserved YEKEK motif of the lepidopteran orthologues as the anchor.

| **Species** | **Accession** | **Length (aa)** | **Equivalent position** | **Residue** | **Sequence group** |
| --- | --- | --- | --- | --- | --- |
| *Thitarodes xiaojinensis* | Chr04.g05884.m1 | 571 | 378 | E | Lepidopteran HSP60 orthologue (reference) |
| *Gynaephora qinghaiensis* | YCJ03831.1 | 572 | 378 | E | Lepidopteran HSP60 orthologue |
| *Hyphantria cunea* | XYO54534.1 | 573 | 378 | E | Lepidopteran HSP60 orthologue |
| *Helicoverpa armigera* | ATB54997.1 | 572 | 378 | E | Lepidopteran HSP60 orthologue |
| *Antheraea pernyi* | APX61067.1 | 575 | 378 | E | Lepidopteran HSP60 orthologue |
| *Trichoplusia ni* | QNN89081.1 | 572 | 378 | E | Lepidopteran HSP60 orthologue |
| *Spodoptera frugiperda* | QNN88711.1 | 573 | 378 | E | Lepidopteran HSP60 orthologue |
| *Mythimna separata* | ATN45247.1 | 568 | 374 | E | Lepidopteran HSP60 orthologue |
| *Grapholita molesta* | ANJ86402.1 | 572 | 378 | E | Lepidopteran HSP60 orthologue |
| *Plutella xylostella* | AIJ00866.1 | 572 | 375 | E | Lepidopteran HSP60 orthologue |
| *Bombyx mori* | XP_062530856.1 | 572 | 378 | E | Lepidopteran HSP60 orthologue |
| *Homo sapiens* | P10809.2 | 573 | 380 | **V** | Vertebrate HSP60 orthologue (HSPD1) |
| *Galleria mellonella* | XP_026760811.3 | 580 | 392 | **N** | 63 kDa mitochondrial chaperonin-like (paralogue) |
| *Manduca sexta* | XP_030024714.2 | 567 | 380 | **S** | 63 kDa mitochondrial chaperonin-like (paralogue) |
| *Drosophila melanogaster* | Q9VMN5.2 | 576 | 380 | E | Hsp60C (paralogue) |

Residue abbreviations: E, glutamic acid; V, valine; N, asparagine; S, serine.

Table S2. The chaperone complement of T. xiaojinensis: family assignment, module membership and co-expression with HSP60A.

The table lists the 63 annotated chaperone genes retained in the co-expression network (11,046 genes × 18 samples), divided into the classical chaperone axis (n = 42: HSP70, HSP90, DnaJ/HSP40, chaperonin TCP-1/cpn60 and one further HSP-family entry) and the small HSP set (n = 21, Hsp20/α-crystallin family, of which 14 form a tandem cluster on chromosome 12). Twelve entries annotated only as chaperone-binding, histone chaperones, tubulin-specific chaperones or copper chaperones are flagged Canonical member = no and were excluded from the classical set; restricting the set to canonical members leaves n = 30. The target gene HSP60A (Chr04.g05884.m1) is shown in the first row. kME = module membership (correlation with the module eigengene); GS (temperature) = Pearson correlation with assay temperature; bicor = biweight midcorrelation with HSP60A computed on log₂(TPM + 1). All network statistics were derived from the signed hybrid network (β = 6; Table S3). ★ = FST-outlier locus identified in the genome-wide differentiation scan (FST = 0.391). Because module membership and connectivity are scale-dependent, the family-level contrasts quoted in the text were verified under log₂(CPM + 1) and variance-stabilised counts as well.

| **Gene ID** | **Family** | **Set** | **Canonical member** | **Module** | **kME** | **kWithin (rank in module)** | **GS (temperature)** | **bicor with *HSP60A*** |
| --- | --- | --- | --- | --- | --- | --- | --- | --- |
| Chr04.g05884.m1 | Chaperonin | classical (target) | yes | turquoise | 0.842 | 4518/4931 | -0.556 | — (self) |
| Chr07.g10434.m1 | HSP40/DnaJ | classical | yes | turquoise | 0.925 | 612/4931 | -0.569 | +0.884 |
| Chr03.g03914.m2 | HSP90 | classical | yes | turquoise | 0.861 | 240/4931 | -0.416 | +0.854 |
| Chr23.g33969.m1 | HSP70 | classical | yes | turquoise | 0.870 | 1601/4931 | -0.709 | +0.850 |
| Chr12.g18441.m2 | Chaperonin | classical | no | turquoise | 0.972 | 72/4931 | -0.663 | +0.845 |
| Chr08.g12182.m1 | Chaperonin | classical | no | turquoise | 0.893 | 4341/4931 | -0.516 | +0.838 |
| Chr07.g10620.m1 | HSP40/DnaJ | classical | yes | turquoise | 0.961 | 552/4931 | -0.558 | +0.825 |
| Chr13.g20503.m1 | HSP40/DnaJ | classical | yes | turquoise | 0.893 | 671/4931 | -0.491 | +0.825 |
| Chr07.g10636.m1 | HSP40/DnaJ | classical | yes | turquoise | 0.921 | 1129/4931 | -0.550 | +0.823 |
| Chr06.g09310.m1 | Chaperonin | classical | no | turquoise | 0.856 | 91/4931 | -0.509 | +0.821 |
| Chr22.g32210.m1 | Chaperonin | classical | no | green | 0.887 | 96/399 | -0.284 | +0.797 |
| Chr32.g43822.m1 | Chaperonin | classical | no | turquoise | 0.831 | 278/4931 | -0.457 | +0.792 |
| Chr03.g04741.m1 | HSP70 | classical | yes | turquoise | 0.912 | 1137/4931 | -0.539 | +0.788 |
| Chr10.g15185.m1 | Chaperonin | classical | yes | turquoise | 0.834 | 2361/4931 | -0.810 | +0.780 |
| Chr13.g19995.m1 | Chaperonin | classical | no | turquoise | 0.731 | 3215/4931 | -0.480 | +0.769 |
| Chr05.g07796.m2 | HSP40/DnaJ | classical | yes | turquoise | 0.887 | 285/4931 | -0.290 | +0.756 |
| Chr01.g01699.m1 | HSP90 | classical | yes | turquoise | 0.889 | 4312/4931 | -0.386 | +0.754 |
| Chr02.g02165.m1 | Chaperonin | classical | no | turquoise | 0.930 | 1159/4931 | -0.615 | +0.748 |
| Chr16.g24747.m1 | Chaperonin | classical | yes | turquoise | 0.879 | 4311/4931 | -0.439 | +0.744 |
| Chr01.g00710.m1 | HSP90 | classical | yes | turquoise | 0.855 | 553/4931 | -0.390 | +0.741 |
| Chr25.g36631.m1 | HSP70 | classical | yes | turquoise | 0.815 | 1885/4931 | -0.590 | +0.721 |
| Chr16.g24393.m1 | HSP90 | classical | no | turquoise | 0.689 | 3040/4931 | -0.119 | +0.707 |
| Chr15.g23884.m1 | Chaperonin | classical | yes | turquoise | 0.816 | 4046/4931 | -0.615 | +0.693 |
| Chr18.g27759.m1 | Chaperonin | classical | no | turquoise | 0.894 | 2504/4931 | -0.413 | +0.675 |
| Chr13.g20555.m1 | HSP40/DnaJ | classical | yes | green | 0.896 | 181/399 | -0.434 | +0.674 |
| Chr02.g02040.m1 | HSP40/DnaJ | classical | yes | turquoise | 0.641 | 3328/4931 | -0.563 | +0.671 |
| Chr10.g14974.m1 | Chaperonin | classical | no | turquoise | 0.849 | 9/4931 | -0.651 | +0.665 |
| Chr09.g14306.m1 | HSP40/DnaJ | classical | yes | turquoise | 0.765 | 4638/4931 | -0.208 | +0.653 |
| novel.1833.1 | Other HSP | classical | yes | green | 0.879 | 227/399 | -0.381 | +0.613 |
| Chr06.g09408.m1 | HSP40/DnaJ | classical | yes | turquoise | 0.831 | 1548/4931 | -0.186 | +0.569 |
| Chr28.g40469.m2 | HSP40/DnaJ | classical | yes | lightcyan | 0.828 | 42/142 | -0.144 | +0.514 |
| Chr18.g27120.m1 | HSP40/DnaJ | classical | yes | turquoise | 0.833 | 2727/4931 | -0.318 | +0.509 |
| Chr22.g33312.m1 | HSP40/DnaJ | classical | yes | blue | 0.759 | 38/612 | -0.392 | +0.503 |
| Chr08.g13315.m1 | Chaperonin | classical | yes | blue | 0.862 | 605/612 | -0.214 | +0.500 |
| Chr12.g18454.m1 | Chaperonin | classical | no | blue | 0.896 | 253/612 | -0.172 | +0.435 |
| Chr11.g16862.m1 | Chaperonin | classical | no | blue | 0.719 | 549/612 | -0.397 | +0.404 |
| Chr25.g36488.m1 | HSP40/DnaJ | classical | yes | green | 0.597 | 317/399 | -0.140 | +0.358 |
| Chr20.g29918.m1 | Chaperonin | classical | yes | grey | -0.125 | 588/623 | -0.177 | +0.131 |
| Chr22.g33011.m1 | HSP40/DnaJ | classical | yes | red | 0.741 | 93/268 | +0.462 | +0.088 |
| Chr01.g01524.m1 | HSP40/DnaJ | classical | yes | greenyellow | 0.252 | 109/222 | +0.408 | -0.099 |
| Chr09.g13399.m1 | HSP40/DnaJ | classical | yes | red | 0.803 | 141/268 | +0.724 | -0.166 |
| Chr13.g20144.m1 | HSP70 | classical | yes | red | 0.814 | 111/268 | +0.705 | -0.203 |
| Chr12.g19373.m1 | sHSP | sHSP | yes | turquoise | 0.590 | 4507/4931 | -0.607 | +0.346 |
| Chr03.g04084.m1 | sHSP | sHSP | yes | cyan | 0.704 | 106/147 | -0.317 | +0.199 |
| Chr12.g19376.m1 | sHSP | sHSP | yes | blue | 0.644 | 410/612 | -0.020 | +0.199 |
| Chr01.g00878.m1 | sHSP | sHSP | yes | green | 0.572 | 288/399 | -0.099 | +0.191 |
| Chr12.g19393.m1 | sHSP | sHSP | yes | grey | -0.153 | 443/623 | -0.015 | +0.126 |
| Chr01.g00891.m1 | sHSP | sHSP | yes | salmon | 0.929 | 1/170 | +0.285 | -0.051 |
| Chr05.g07857.m1 | sHSP | sHSP | yes | royalblue | 0.316 | 91/93 | +0.089 | -0.170 |
| Chr18.g26761.m1 | sHSP | sHSP | yes | grey | -0.199 | 503/623 | -0.142 | -0.183 |
| Chr12.g19372.m1 | sHSP | sHSP | yes | grey | 0.034 | 522/623 | -0.248 | -0.257 |
| Chr01.g00889.m1 | sHSP | sHSP | yes | grey60 | 0.727 | 6/114 | +0.116 | -0.276 |
| Chr12.g19371.m1 | sHSP | sHSP | yes | yellow | 0.544 | 254/460 | +0.554 | -0.356 |
| Chr18.g27722.m1 | sHSP | sHSP | yes | cyan | 0.329 | 52/147 | -0.093 | -0.389 |
| Chr12.g19377.m1 | sHSP | sHSP | yes | grey | 0.325 | 580/623 | +0.294 | -0.396 |
| Chr12.g19390.m1 | sHSP | sHSP | yes | black | 0.537 | 55/257 | +0.569 | -0.472 |
| Chr12.g19388.m1 | sHSP | sHSP | yes | grey | 0.790 | 447/623 | +0.590 | -0.498 |
| Chr12.g19385.m1 | sHSP | sHSP | yes | black | 0.386 | 105/257 | +0.496 | -0.584 |
| Chr12.g19381.m1 | sHSP | sHSP | yes | black | 0.623 | 143/257 | +0.525 | -0.592 |
| Chr12.g19383.m1 | sHSP | sHSP | yes | black | 0.610 | 249/257 | +0.550 | -0.614 |
| Chr12.g19389.m1 | sHSP | sHSP | yes | grey | 0.692 | 598/623 | +0.639 | -0.623 |
| Chr12.g19387.m1 | sHSP | sHSP | yes | grey | 0.724 | 327/623 | +0.717 | -0.625 |
| Chr12.g19379.m1 | sHSP | sHSP | yes | black | 0.563 | 54/257 | +0.607 | -0.642 |

Table S3 Soft-threshold selection for scale-free network construction (signed hybrid adjacency, 11,046 genes × 18 samples). Values are the signed scale-free topology fit R² and the mean connectivity computed by WGCNA. No tested power met the conventional R² ≥ 0.85 criterion; the fit reached 0.81 only at β = 20, by which point mean connectivity had fallen to 19.8 and module sizes had contracted sharply. β = 6 (row highlighted) was therefore used for network construction.

| **β** | **Signed R²** | **Mean connectivity** | **R² ≥ 0.85 met?** |
| --- | --- | --- | --- |
| 1 | 0.11 | 2,833.3 | No |
| 2 | 0.60 | 1,460.5 | No |
| 3 | 0.70 | 879.3 | No |
| 4 | 0.71 | 579.5 | No |
| 5 | 0.73 | 405.3 | No |
| 6 | 0.75 | 295.6 | No |
| 7 | 0.75 | 222.6 | No |
| 8 | 0.76 | 171.7 | No |
| 9 | 0.76 | 135.1 | No |
| 10 | 0.76 | 108.1 | No |
| 12 | 0.78 | 72.0 | No |
| 20 | 0.81 | 19.8 | No |
